# Metabolic activation of 2,4,6-trinitrotoluene; a case for ROS-induced cell damage

**DOI:** 10.1016/j.redox.2024.103082

**Published:** 2024-02-15

**Authors:** Amma Gyapomah Adomako-Bonsu, Jana Jacobsen, Edmund Maser

**Affiliations:** Institute of Toxicology and Pharmacology for Natural Scientists, University Medical School Schleswig-Holstein Campus Kiel, Brunswiker Str. 10, 24105, Kiel, Germany

**Keywords:** Submerged munitions, 2,4,6-Trinitrotoluene (TNT) toxicity and carcinogenicity, Metabolic activation, Marine ecotoxicity, Human toxicity

## Abstract

The explosive compound 2,4,6-trinitrotoluene (TNT) is well known as a major component of munitions. In addition to its potential carcinogenicity and mutagenicity in humans, recent reports have highlighted TNT toxicities in diverse organisms due to its occurrence in the environment. These toxic effects have been linked to the intracellular metabolism of TNT, which is generally characterised by redox cycling and the generation of noxious reactive molecules. The reactive intermediates formed, such as nitroso and hydroxylamine compounds, also interact with oxygen molecules and cellular components to cause macromolecular damage and oxidative stress. The current review aims to highlight the crucial role of TNT metabolism in mediating TNT toxicity, via increased generation of reactive oxygen species. Cellular proliferation of reactive species results in depletion of cellular antioxidant enzymes, DNA and protein adduct formation, and oxidative stress. While TNT toxicity is well known, its ability to induce oxidative stress, resulting from its reductive activation, suggests that some of its toxic effects may be caused by its reactive metabolites. Hence, further research on TNT metabolism is imperative to elucidate TNT-induced toxicities.

## Graphical abstract

**Figure:**
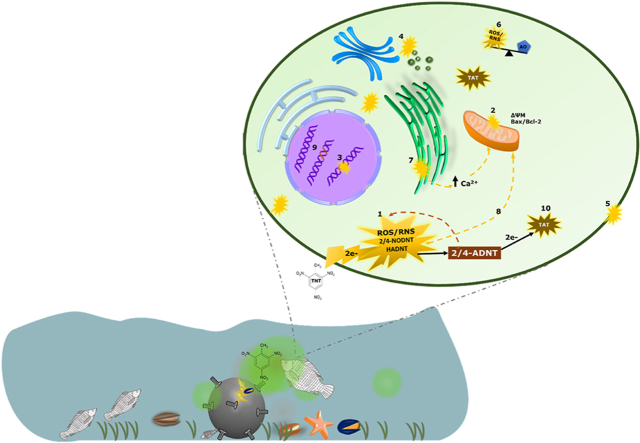

## 1 Introduction

The explosive compound 2,4,6-trinitrotoluene (TNT) is well known due to its use as a principal constituent of munitions. Indeed, some of the significant hazards caused by war residues and munitions have been traced back to the early-20th century, during World Wars I and II. In 1918, 7000 to 17,000 cases of poisoning (including some fatalities) and 181 jaundice cases were reported among workers in American and English shell-loading plants, following their exposure to TNT; a major component of the million tons of cannon ammunitions produced for the European war [1]. Apart from the socioeconomical impacts of wars, which persist, the environmental impact of wars has recently gained immense public attention. For example, it has become apparent that the excess munitions dumped into the seas after both World Wars are an ongoing threat to the marine ecosphere and human anthropogenic activities such as fishing and recreation. Significant evidence also suggests that munition-derived nitroaromatic compounds such as TNT are leaking into the seas. Munitions dumpsites in the Baltic Sea region (such as the Kolberger Heide -Kiel and the Luebeck Bay -Luebeck), in particular, are contaminated with explosive compounds, with detection of TNT and its main metabolites (2-amino-4,6-dinitrotoluene (2-ADNT) and 4-amino-2,6-dinitrotoluene (4-ADNT)) in sediments and marine fauna [2,3]. More so, the occurrence of TNT and its metabolites in seafood has been well documented [[4], [5], [6], [7]]. Although current scenarios do not indicate risks to the human seafood consumer [8], continuous TNT leakage from sea-dumped munitions could pose a threat to humans in the future, since TNT carcinogenicity and mutagenicity is well known [9]. Oral exposure to TNT via consumption of contaminated crop leaves (such as fruits and cereals) that had been cultivated at former military sites in Vieques (where unsafe levels of ammunition residues were recorded after decades of military activities) led to bladder cancer in humans [10].

In spite of their harmful effects, nitroaromatics continue to find relevance in the production of pharmaceuticals, pigments, polymers, and pesticides, at over one hundred million tons annually [[11], [12], [13], [14], [15], [16]]. Particularly, TNT is regarded as an economical alternative in industrial and chemical engineering sectors, where direct exposure to human workers (via inhalation and skin contact) and the environment (via waste disposal) is inevitable. Entry of TNT and 2/4-ADNT into ground and underground waters from contaminated soils and wastewater from abandoned TNT plants [17], and fresh-water lakes, from dumped munitions [18], has also been reported. Therefore, the apparent ubiquitous occurrence of TNT in the environment increases the potential for both short-term and long-term toxicities in humans and animals.

Toxicity of TNT and, to some extent, its principal metabolites 2/4-ADNT is a well-researched topic. A quick search in PubMed for articles published within the past 20 years, using the search terms ‘2,4,6-Trinitrotoluene and toxicity’, generates at least 890 results (2,4,6-Trinitrotoluene and toxicity - PMC - NCBI (nih.gov)). One of these studies has assessed the risk of human exposure to these explosive compounds, via intake of contaminated sea food [5]. At the cellular level, it is generally agreed that biochemical events such as oxidative stress (OS), and DNA damage play a key role in the mechanism of TNT toxicity. Enzyme-catalysed redox cycling of TNT, as well as protein and DNA adduct formation by its reactive intermediates, could mediate cytotoxic effects of TNT. However, despite the several reports on TNT toxicity and its induction of OS, the causative role of TNT-mediated OS in TNT toxicity has not been sufficiently highlighted. OS induction can be demonstrated by a plethora of experimental assessments, including increased generation of free radicals (reactive oxygen species -ROS, reactive nitrogen species -RNS, reactive sulfur species -RSS, and reactive metabolites) and the subsequent depletion of cellular antioxidants (which exacerbates ROS accumulation and macromolecular damage), lipid peroxidation and DNA oxidation.

This review focuses on this gap in literature by presenting the evidence for TNT-mediated OS and its association with TNT toxicities reported. The Sections in this article focus on the avenues of ROS generation from the TNT metabolism pathway and the effects of TNT and some of its metabolites on OS biomarkers. In keeping with this aim, reactive intermediates or OS triggers from the TNT metabolism pathway, rather than enzymes involved in TNT metabolism, are presented in detail with evidence from existing reports. This review will also discuss the causative role of TNT metabolites in TNT toxicities while highlighting the existing research gaps on the fate of the principal (and seemingly stable) metabolites of TNT such as 2/4-ADNT in cells or organisms. However, the scope of this review excludes the general mechanism of ROS-induced oxidative stress, antioxidant stress response, or a compilation of the toxic effects of TNT.

## 2 Chemistry and metabolism

TNT is a nitroaromatic energetic compound with three nitro groups, which are substituted at the *ortho* and *para* positions of the methyl group on the benzene ring. TNT is regarded as a common mass explosive not only because of its explosive power but also due to its good chemical and thermal stability, enabling quite safe production and storage. In addition, TNT has a fairly low melting point of 80 °C, which facilitates its pouring process. Due to its poor solubility in water (130 to 150 mg/L) (Agency for Toxic Substances and Disease Registry, 1995; [19]), as well as its low vapour pressure (7.2 × 10-9 atm) [19] and an octanol-water coefficient of 1.86 [19], it can be assumed that dissolved TNT is not irreversibly bound to sediments. Reversible sorption at the bottom via hydrogen bonds and ion exchange, on the other hand, is known [20]. Its physical properties suggest that mobility within an entire environment is possible. Although reduction of the nitro groups has been shown to increase the occurrence of irreversible sorption [21].

In addition to by-products from its production, biotic and abiotic transformation products of TNT can also be detected in the environment. Both oxidative metabolism with reaction products such as trinitrobenzylalcohol, trinitrobenzoicacid, and trinitro-m-cresol, and reductive metabolism have been observed in animal experiments [22]. In reductive metabolism, the two metabolites 2-amino-4,6-dinitrotoluene (2-ADNT) and 4-amino-2,6-dinitrotoluene (4-ADNT) are of relevance, where 4-ADNT is the main metabolite [4,23]. These compounds are formed by metabolic or abiotic reduction of one of the three nitro groups to an amino group [24,25]. This first reduction step converts the electron-withdrawing nitro group into an electron-donating amino group and thus the electron density distribution changes. The remaining nitro groups gain electron density, which makes further reduction of the molecule more difficult. This also shows that the diamino products 2,4-diamino-6-nitrotoluene (2,4-DANT) and 2,6-diamino-4-nirotoluene (2,6-DANT) could be detected to a lesser extent in tissues [26].

**Fig. 1:**
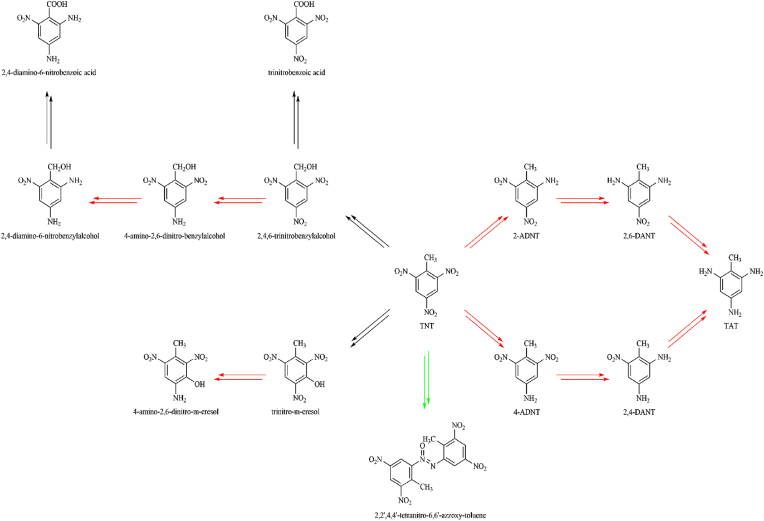
Chemical structures of TNT and its principal metabolites (red: reduction, black: oxidation, green: dimerization).

Metabolic reductions of the nitro compounds occur via a two-electron reduction to the nitroso and hydroxylamine intermediates and then to the amine, catalysed by mammalian NAD(P)H quinone dehydrogenase 1 (NQO1)[27]; or via a one-electron reduction, which leads to the formation of anion-free radical intermediates (see Fig. 3; [28]). One-electron reduction to nitro radical anions, catalysed by flavoenzyme dehydrogenase electrotransferases such as NADPH: cytochrome P450 reductase (EC 1.6.2.4) [[29], [30], [31]], ferredoxin:NADP^+^ reductase (FNR, EC1.18.1.2) [27,30] and bacterial oxygen-sensitive nitroreductases (under aerobic condition), leads to redox cycling and ROS generation. ROS formation resulting from TNT metabolism is discussed in detail in the next Section. Single-electron reduction of nitroaromatics is believed to underpin their cytotoxic effects and is largely influenced by their single-electron reduction potential [[32], [33], [34], [35], [36]]. The single-electron reduction potential (E^1^_7_ value) of nitroaromatics is determined by their rate of interaction with single electron transferring enzymes [27,29,30]. This also depends on their nucleophilic substituents and could be useful in ascertaining the extent of OS involvement in cytotoxicity by nitroaromatics [27]. Fig. 1 shows the chemical structures of TNT and its principal metabolites, and key chemical process they undergo during metabolism.

## 3 Generation of reactive species during TNT metabolism

**Fig. 2:**
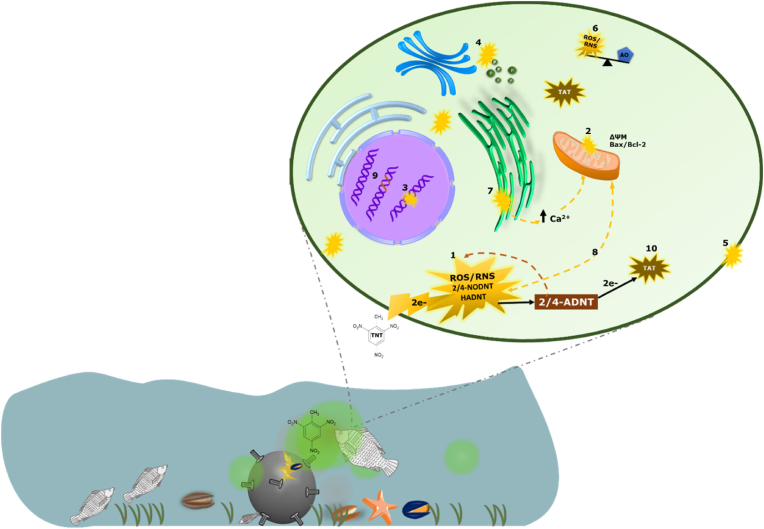
Graphical representation of ROS damage following TNT metabolism activation; as reported in literature (see details in text).

Metabolism of nitroaromatics such as TNT can be regarded as prooxidative, yielding metabolites that are unstable and can promote ROS generation. This involves sequential reduction of the parent nitroaromatic compound to nitroso and hydroxylamine intermediates, both of which undergo redox cycling to cause OS [12]. As previously mentioned, the involvement of mitochondrial nitroreductase and microsomal cytochrome P450-NADPH reductase in one- or two-electron reduction of nitroaromatic compounds inevitably leads to the formation of free radicals. Free radicals, including reactive nitrogen species (RNS - ^•^NO, ^•^NO_2_, and ONOO−), reactive oxygen species (ROS - O_2_^•−^, H_2_O_2_, ^•^OH, RO_2_^•^, RO^•^, ^1^O_2_, and O_3_) and reactive sulfur species (RSS) are ubiquitous in cells, serving as key signalling molecules [37]. Cellular ROS is primarily generated during mitochondrial respiration and as by-products of oxygen-dependent enzymatic reactions by CYP P450 and plasma membrane bound NADPH oxidase (NOX) [37]. Therefore, the generation of excess free radicals during TNT metabolism, and the presumed (but indisputable) interaction of unstable intermediates with oxygen molecules overwhelms cellular antioxidant defences, leading to OS (see (1) in Fig. 2).

**Fig. 3:**
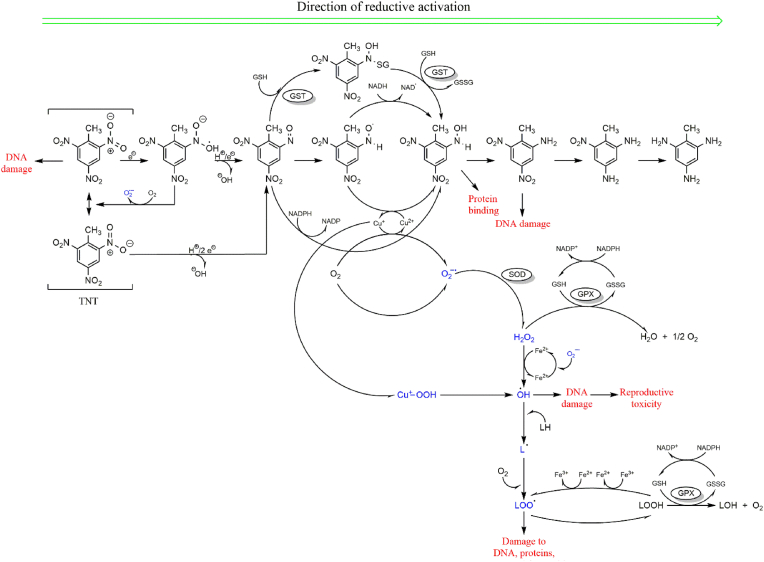
Schematic representation of free radical generation processes during TNT metabolism. Single- or double-electron reduction of TNT results in the formation of reactive nitroso radicals, which initiate a cascade of ROS generating events. Persistent ROS generation and accumulation lead to macromolecular damage and other organ toxicities, as reported in subsequent Sections.

The concept of ‘ROS formation as a consequence of the reductive activation of TNT’ by nitroreductases was initially proposed by Zitting et al. [38]. In their study, significant increase in superoxide (O_2_^•−^) levels were recorded in isolated mitochondria and microsomes after TNT treatment. Indeed, the strong electronegativity of its substituent nitrogen and oxygen atoms renders the TNT aromatic ring unstable (or polarised). Thus, reduction of these electrophilic nitro groups to their respective nitroso metabolites marks the initial step in TNT metabolism. Even though several points for ROS leakage exist in the TNT metabolism pathway (see Fig. 1, Fig. 3), the sequential reduction of the nitro substituents of the parent compound and subsequent intermediates also influences redox homeostasis.

Following exposure of marine biota to TNT-containing munitions, TNT undergoes single-/double-electron reduction to form reactive metabolites and well-known cellular free radicals (1). Details on the redox processes and type of free radicals formed during TNT reduction have been presented in Fig. 3. These reactive species attack the mitochondria (2), DNA (3), peroxisomes (4), cell membrane (5) and cause cellular redox imbalance (6). Additional effects include intracellular calcium overload due to ER attack (7), redox cycling due to mitochondrial ROS release (8) and mutagenicity/chromosomal aberration (9), and formation of further reactive metabolites from the more stable principal metabolites (2/4-ADNT; 10).

Interestingly, the predominant contributors of TNT-induced ROS/RNS generation seem to be formed from the one-electron reduction steps that precede 2/4-ADNT formation. Metabolic activation of TNT via one-electron transfer to form nitroso radical anion, and hydroxylamine and amine in subsequent steps is clearly conserved across species [39]; resulting in ROS accumulation and ultimately OS [40]. Single-electron transfer during TNT biotransformation is also known [16,[40], [41], [42]], and this is reflected in the fate of TNT in eukaryotic cells.

One-electron reduction catalysed by NOS (nitric oxide synthase) in a CaCl_2_/calmodulin-dependent reaction was also posited by Kumagai et al. [43] as a pivotal step in the generation of TNT nitro radicals (also considered as competent reactive nitrogen radicals, RNS). One-electron reduction of 2/4-nitroso dinitrotoluene yields the transient but reactive metabolite 2/4-hydroxylaminodinitrotoluene (see Fig. 3; [44]). These RNS exacerbate redox imbalance by reacting with molecular oxygen to form O_2_^•−^ and hydrogen peroxide (H_2_O_2_) [43]. Reduction of TNT by nitroreductases also produces transient and unstable hydroxylamine intermediates, which undergo reoxidation by oxygen, isomerisation to amino phenols or formation of azoxy derivatives by reacting with nitroso compounds [27].

Mammalian NADPH:quinone oxidoreductase [27,28,[45], [46], [47]] and bacterial oxygen-insensitive nitroreductases catalyse two-electron reduction of nitroaromatics to nitroso compounds and subsequently to alkylating hydroxylamines [48]. For TNT, this type of reduction occurs on either of its *ortho* or *para* nitro substituents to form 2-nitroso-4-dinitrotoluene or 4-nitroso-2-dinitrotoluene, respectively (Fig. 3). This step also releases ^•^OH radicals, contributing to intracellular redox imbalance. ^•^OH is notorious for its ferocious attack on cellular macromolecules, especially on lipids, to initiate and propagate peroxidation of cell membrane components. Furthermore, reduction of the remaining nitro substituents to reactive intermediates and relatively stable metabolites such as 2/4-ADNT provides an avenue for leakage of reactive molecules, exacerbating surging cellular ROS levels.

ROS generation as a consequence of TNT metabolism has been observed both *in vivo* and *in vitro*. O_2_^•−^ generation was observed in cultures of *Bacillus cereus* ZS18 and *Pseudomonas* sp ENE1582 [49], callus Tartary buckwheat [50], and *Yarrowia lipolytica* AN-L15 [51] after TNT reduction. Analysis of ROS generation in liver, brain and kidney cortex and medullae, following TNT exposure, showed increased levels of O_2_^•−^; H_2_O_2_ was predominantly high in rat liver [52]. In addition, their comparative study on liver microsomes and mitochondria from rats (*in vitro*) and monkeys (*in vivo*) showed a concentration-dependent increase in O_2_^•−^ and H_2_O_2_ generation following TNT exposure. Liao et al. also reported increased ROS and mitochondrial membrane potential (see (2) in Fig. 2) in HepG2 cells, which are of course liver derived [53]. Undoubtedly, the intensity of TNT-induced ROS generation is largely defined by its metabolism, as observed in rat liver and kidney samples (high H_2_O_2_ levels were detected) [52]. TNT also interacts with the cytochrome P450 reductase domain of neuronal NOS (nNOS) to usurp electrons from NADPH (an activity that is facilitated by NADPH oxidase activation by TNT), resulting in O_2_^•−^ formation [54]. More so, high ROS levels recorded after 15 min of TNT exposure (100 μM) in bovine aortic endothelial cells emphasise the involvement of transient reactive metabolites (see Fig. 3) in redox imbalance [54].

Although the electron reduction potential (E^1^_7_ value) of TNT does not influence its reduction by NQO1, this reaction also contributes significantly to TNT-induced ROS generation [27]. Furthermore, O_2_^•−^ radicals formed can also react with NO (NO is synthesised by NO synthase) to form nitrogen dioxide radicals (^•^NO_2_). The reactivity of these radical species with macromolecules affects cell function and viability. This is discussed in the subsequent Sections.

## 4 ROS mediate toxicity of TNT and its metabolites

Compared to the number of reports on the toxicity of TNT and some of its known metabolites, fewer studies have confirmed the role of OS in mediating these toxicities. What seems rather apparent is that majority of these toxicity studies were designed to identify the presence and, subsequently, effects of TNT and its metabolites in the experimental models used, rather than identifying the fundamental cause of the toxicities observed. For instance, seven years after Zitting et al. observed clumping of nuclear chromatin and mitochondrial swelling in proximal tubulin of intraperitoneally-treated male Wistar rats (100 mg/kg TNT; [38]), Kong et al. reported on the role of increased ROS formation in the mitochondria of kidney cortex (due to reductive activation of TNT) in mediating those toxicities ([52]; see (2) in Fig. 2). The cytotoxic effects of nitroaromatics such as TNT generally correlates positively with their single-electron reduction potentials and lipophilicities [27]. This is typical of oxidative stress-mediated cytotoxicity [55].

Reproductive toxicity caused by surging ROS levels was also observed in male Fischer 344 rats, with significant histological damage and testicular and epidydimal atrophy following subacute exposure to TNT [56]. Accumulation of the N-hydroxy TNT intermediate 4-hydroxylamino-2,6-DNT (HADNT) as well as its reduction to ADNT resulted in oxidative DNA damage (see (3) in Fig. 2) due to 8-oxo-7,8-dihydro-2'-deoxyguanosine (8-oxodG) and ^•^OH formation in sperms, and 8-oxodG accumulation in the caput epididymis and calf thymus DNA. Furthermore, significant reduction in spermatozoa and atrophy of the testis and epididymis were also observed [56]. Here, the TNT intermediate HADNT, but not TNT, caused oxidative DNA damage at serial guanine residues.

In an *in vitro* study, 3-h incubation of mammalian (rat, mouse and human) liver cells with 10 mg/ml TNT resulted in significant increase in cellular ROS levels associated with peroxisome proliferation (see (4) in Fig. 2) and apoptotic nuclear morphology [57]. Sun et al. [54] observed endothelial NOS (eNOS) phosphorylation via H_2_O_2_-mediated P3I/Akt activation in TNT-treated bovine aortic endothelial cells. However, inhibition of eNOS activity in bovine aortic endothelial cells by TNT disrupted NO formation and caused dose-dependent hypertension in TNT-treated rats [58]. Thus, increased ROS generation could be key in mediating vascular effects of TNT. Moreover, increased O_2_^•−^ and ^•^OH generation after TNT treatment in *Fagopyrum tartaricum* callus cells led to cell membrane damage via lipid peroxidation [50]. Three-week old *Arabidopsis thaliana* seedlings also showed DNA tailing after 24-h exposure to 10 mg/kg TNT; this effect was preceded by O_2_^•−^ accumulation at 15 h [59].

Furthermore, generation of ^•^OH during TNT metabolism supports the ferocious nature of TNT-induced OS, given that ^•^OH attacks cellular lipid molecules, including membrane lipids. This disrupts cellular structure and function. TNT caused a 3.8-fold increase in malondialdehyde (see (5) in Fig. 2) after 24 h treatment and this resulted in 85–90% cell death in lamb kidney fibroblasts, which was partly averted by antioxidants N,N’-diphenyl-p-phenylene diamine and desferrioxamine; LD_50_ = 25 ± 5.0 μM [27]. Toxicity of amino and hydroxylamino metabolites of TNT also occurs via flavoenzyme-catalysed redox cycling, where these metabolites serve as substrates for single-electron transfer [60] to generate more reactive molecules.

## 5 Interactions with cellular OS biomarkers

In addition to surging cellular ROS levels, the up-regulation and/or down-regulation of prominent stress response genes and proteins aptly serve as prominent indicators of oxidative stress. Redox homeostasis is maintained by cellular defence mechanisms, comprising a complex network of short- and long-acting direct and indirect antioxidants (see reviews: [[61], [62], [63], [64]]. Changes in the expression of cellular antioxidant enzymes such as catalase, superoxide dismutase (SOD), Kelch-like ECH Associated Protein 1 (Keap1)/nuclear factor erythroid 2-related factor 2 (Nrf2)/antioxidant response element (ARE) complex, and the glutathione/glutathione disulfide (GSH/GSSG) ratio have been reported as indicators of OS events [61,63,65,66], considering their role in attenuating ROS-induced effects.

### 5.1 Induction of direct-acting stress response enzymes

Induction of stress response enzymes, such as carbonyl reductase, highlights the complexity of the oxidative stress/carbonyl stress phenomena. This has been recently identified as a relevant biomonitoring marker for the assessment of TNT levels/effects at contaminated sites [3,6,44]. Moreover, the nature of toxicities caused by xenobiotic exposure could largely be influenced by the antioxidant capacity of the cell or organism being examined. Most organisms respond adequately to surging ROS levels. Certainly, the initial generation of O_2_^•−^ presents the basis for subsequent formation and accumulation of more atrocious ROS such as H_2_O_2_ and ^•^OH (hydroxyl radical). Dismutation of O_2_^•−^ results in the formation of H_2_O_2_ (substrate for Fenton reaction), significant levels of which were also recorded after TNT exposure [54,67]. Hence, antioxidant-deficient organisms or cells are often more susceptible to oxidative damage. For instance, the nominal expression of SOD (a O_2_^•−^ dismutation enzyme) in bovine lenses augmented O_2_^•−^ accumulation and OS during TNT reduction by zeta-crystallin, leading to cataract formation [68]. Oxidative stress is a known trigger for cataracts [69,70], which has also been observed after chronic TNT exposure to humans [[71], [72], [73], [74], [75], [76]].

Furthermore, up-regulation of SOD and catalase mRNA and protein expression was observed in TNT-treated mouse (Hepa 1–6) and human (HepG2) liver cells [57]. Both cell lines, as well as rat liver cells, also showed peroxisome proliferation; possibly due to TNT-induced ROS generated during exposure [57]. In Gram-positive *Bacillus pumilus* GY, TNT exposure triggered up-regulation of catalase and the thioredoxin/thioredoxin reductase system [67]. Tchounwou et al. [77] previously observed approximately 80% decline in cell viability after 48-h exposure to 300 μg/ml TNT. This correlated positively with significant gene induction of cytochrome P450 subfamily 1A, glutathione S-transferase Ya, and xenobiotic response element in HepG2 cells [77]. These known phase I and II biotransformation genes could undoubtedly be involved in the metabolism of TNT to radical species. Hence, one can infer that reactive TNT metabolites could exacerbate redox imbalance and ultimately cause oxidative stress.

### 5.2 TNT induces Nrf2 stress response complex

Activation of Nrf2, the master transcription factor for stress response, was also observed in the gene network of rat liver treated with the TNT metabolite 2,4-DNT [78]. This leucine zipper transcription factor regulates the expression of a battery of cytoprotective genes such as NQO1, heme oxygenase-1, thioredoxin, glutathione S-transferase, glutathione peroxidase, and glutamine-cysteine ligase [62]. Induction of this pathway is mainly via interaction of inducers such as electrophiles and ROS (see Ref. [61] with the Keap1 cysteine (Cys) residues, which act as regulators of Nrf2 intracellular stability (Nrf2 undergoes Keap1/Cullin-3 (Cul3)-based E3 ligase ubiquitination and proteasomal degradation in the absence of inducers). Reactive free radicals such as RNS and ROS mainly interact with Keap1, causing inter- or intra-molecular (or interdomain) disulfide bonds between cysteine residues in the Brand complex, Tramtrack and Bric a brac (BTB), intervening region back (IVR), and C-terminal domains (CTR) [79,80]. Fourquet et al. [79] observed oxidation of Keap1 Cys^151^ (in the BTB domain) by NO, leading to prolonged stabilisation (and, therefore, nuclear translocation) of Nrf2 in Hela cells. The Keap1-Keap1 disulfide linkage observed (via Cys^151^) implies that the resulting Keap1 deficiency, in addition to Nrf2 availability, promotes Nrf2 accumulation, phosphorylation and (eventually) nuclear translocation [79]. Furthermore, alteration at Cys^151^ leads to conformational changes in the Keap1 BTB, decreasing the affinity of Keap1 for Cul3 [81,82]. This affects Nrf2 repression and ubiquitination by Keap1 and Cul3, and promotes Nrf2 nuclear accumulation. Nuclear translocation of Nrf2 in response to OS is also triggered by ROS interaction with Keap 1 cysteine residues in the CTR domain [83]. In Keap1 mutants (*in vivo* -murine and *in vitro* -mouse embryonic fibroblasts) lacking selected Cys residues, H_2_O_2_ induced conformational change via intramolecular disulfide formation between Cys^226^ (IVR domain), Cys^613^ and Cys^622/624^ (CTR domain) to promote Nrf2 stability and accumulation [83]. GSSG formed during OS also activate Nrf2 via intermolecular disulfide formation at Cys^319^ of the Keap1 IVR domain [84]. Cys^151^, Cys^273^ and Cys^288^ are also indispensable for Nrf2 activation by 4-hydroxy-2-nonenal [85]. The occurrence of increasing ROS, such as H_2_O_2_, has been observed after TNT exposure [54].

Although TNT may not be a typical Nrf2 inducer, reports on its effects on this transcription factor highlight a rather complex oxidant propensity (for TNT) under metabolic conditions. Considering the generous formation of diverse RNS during TNT reduction (see Fig. 3), persistent induction of Nrf2 could be postulated. The redox instability of NO in the presence of oxygen undoubtedly positions it as an additional source for RNS/ROS formation, perpetuating Nrf2 activation; via nitrosylation by NO-related reactive species (such as N_2_O_3_ and ^•^NO_2_) and transnitrosation of vicinal Cys residues jo [[86], [87], [88], [89], [90]]. Accordingly, Fourqet et al. reported persistent Nrf2 induction in the presence of NO, but not H_2_O_2_ (which is susceptible to cellular thiol reductases such as GSH and thioredoxin) [79].

In the nucleus, Nrf2 complexes with small musculoaponeurotic fibrosarcoma (sMaf) transcription factors to form a heterodimer. This Nrf2/sMaf heterodimer activates phase 2 enzymes by binding to ARE in their upstream regulatory region. Thus, phosphorylation of Nrf2 exerts cytoprotective effect in the presence of oxidant and electrophile toxicity [91]. Genes of the Nrf2 stress response pathway, which correspond to downstream phase I and II metabolizing enzymes such as NQO1, aldo-keto reductase 7A3, UDP-glucuronosyltransferase 1A6, glutathione transferases and epoxide hydrolase 1, were modulated by TNT, 2,4/6-DNT, and 2/4-ADNT in rat hepatocytes [78]. An earlier study in Northern bobwhite liver also revealed Nrf2 induction by 2,6-DNT [92].

### 5.3 TNT induces protein modification

More so, concentration–dependent induction of the gene for heat shock protein 70 (Hsp70) was recorded in response to TNT [77]. Hsp70 is an essential chaperone for protein folding and repair, and maintenance of cellular proteostasis [93]. In their *in vivo* study with TNT-treated female Sprague-Dawley rats, Deng et al. [78] also observed a time-dependent up-regulation of *hsp* upon gene ontology (GO) enrichment analysis of their liver samples [78]. It was ascertained that these effects resulted from cellular activities of reactive intermediates [78]. This certainly agrees with time-dependent increase in TNT-induced free radical generation, which occurs during its metabolism. Under OS condition, ROS recruit Hsp70 via oxidation of its Cys residues [94] and this leads to reduction of ROS levels [95,96]. In addition to promoting the degradation of oxidized proteins and protein aggregates, Hsp70 exerts cytoprotective effects by inducing antioxidant enzymes such as glutathione peroxidase and glutathione reductase [94,95].

Enrichment in up-regulated differentially expressed proteins also revealed induction of stress response proteins, such as glutathione S-transferase in TNT-treated Arabidopsis [59]. In a recent report, down-regulation of glutathione S-transferase and catalase (see (6) in Fig. 2) occurred following a 28-day exposure of the soil annelid *Eisenia fetida* to 10.6 and 38.7 mg TNT/kg soil [97]. In addition, methemoglobinemia (resulting from one-electron reduction of TNT by hemoglobin) caused altered oxygen binding and transport, and down-regulation of ferritin due to increased Fe^3+^ oxidation and Fe^2+^ depletion [97]. Ferritin maintains cellular iron balance (via rapid detoxification, mineralisation and storage of iron); this mitigates ROS formation [98,99]. Its cytoprotective activity could also be facilitated by the presence of ARE in both H and L subunits. Therefore, down-regulation of ferritin following TNT exposure (as reported in soil annelids) could signal persistent ROS accumulation, promote iron imbalance, and exacerbate OS. In murine erythroid cells [100] and Hela cells [101], overexpression of ferritin H (this subunit exhibits a high ferroxidase activity) resulted in protection against H_2_O_2_. Furthermore, methemoglobinemia has been reported as a consequence of human exposure to TNT, see Section 7.

### 5.4 TNT-induces calcium dyshomeostasis

Interactions between TNT and the inner mitochondrial membrane also generate ROS via NADP-NADPH redox cycling and Ca^2+^ dyshomeostasis [97]. Intracellular Ca^2+^ dyshomeostasis is a major event that is capable of exacerbating oxidative damage due to its ability to subsequently trigger high ROS production in what has been labelled “Ca^2+^-ROS crosstalk” by Peng and Jou [102]. ROS such as O_2_^•−^ and H_2_O_2_ [103] can cause Ca^2+^ release from the endoplasmic/sarcoplasmic reticulum (ER/SR) by: 1) oxidizing the sulfhydryl groups of sarco-endoplasmic reticulum Ca^2+^-transporting ATPases (SERCA); 2) directly interacting with their ATP-binding site; or 3) modulating inositol 1,4,5-triphosphate (InsP3) levels, InsP3-operated channels and ryanodine receptors [[104], [105], [106]]. Elevated intracellular Ca^2+^ concentration (see (7) in Fig. 2) impairs cellular organelles, including the ER (accumulation of modified proteins due to defective chaperone activity worsens ROS generation in a regulatory mechanism), nucleus (activation of endonucleases for apoptotic cleavage and fragmentation of chromatin and nuclear proteins), and mitochondrion (due to its proximity to the ER, the mitochondrion encounters relatively high Ca^2+^ levels) [107]. Moreover, mitochondria Ca^2+^ influx alters properties of the inner mitochondrial membrane by disrupting transmembrane potential [[108], [109], [110], [111], [112], [113], [114]], and interacting with anionic heads of cardiolipin (an abundant component of the inner membrane) to displace cytochrome *c* [115]. The resulting boost in oxidative phosphorylation and ATP synthesis (requiring high oxygen consumption), in addition to delays at complexes III and IV, promote the leakage of electrons from the ETC and the formation of semiquinones and O_2_^•−^ radicals (in the presence of inorganic phosphate) [115,116]. Therefore, in addition to the noxious and diverse free radicals generated by TNT metabolism, ROS/Ca^2+^-mediated increase in mitochondrial ROS production, opening of mitochondrial permeability pores and mitochondrial swelling, and the escape of ROS-detoxification proteins such as cytochrome *c*, GSH (and NADPH, a relevant cofactor for the restoration of antioxidants such as glutathione reductase and glutathione peroxidase) from the intermembrane space aggravate TNT-induced OS; and cellular damage as a result. Increased ROS generation in TNT-treated HepG2 cells led to mitochondrial dysfunction, loss of mitochondrial membrane potential and activation of caspases [53]. Furthermore, ROS-induced ROS generation (see (9) in Fig. 2), which can also evidently result from inhibition of respiration at complex IV by RNS such as NO^−^ and ONOO^−^ could advance OS; reduction of TNT produces highly reactive intermediate nitrogen radicals (see Fig. 3). This emergence of redox cycling and intracellular Ca^2+^ imbalance from TNT metabolism is not atypical of toxicity by nitroaromatics. For instance, hepatotoxicity by nilutamide, a nonsteroidal antiandrogenic nitroaromatic drug, is mediated by its metabolism (leading to the formation of nitroso radicals) and redox cycling, hepatic ROS overload, intracellular Ca^2+^ surge and OS [117]. Similarly, nitroreduction of nimesulide (a selective cyclo-oxygenase 2 inhibitor) via one-/two-electron transfer results in nitroso radicals and aromatic amine derivatives [118]; which undergo redox cycling to exacerbate redox imbalance, causing NAD(P)H oxidation, protein modification, calcium-mediated opening of mitochondrial permeability transition pores and mitochondrial damage [119]. These effects, in addition to OS, underly nimesulde-induced hepatotoxicity [120].

### 5.5 TNT upregulates carbonyl reductase expression

Recently, transplanted mussels (*Mytilus* spp.) exposed at the Kolberger Heide at the Kiel Fjord in Germany (an area well known for the uncovering of dumped munitions [3,4,[121], [122], [123]] for 58 days showed up-regulation of the carbonyl reductase gene [124]. This finding was in congruence with the presence of the explosive compound TNT and ADNT in the exposed mussels. A subsequent lab-based investigation on the induction of this stress response gene by TNT revealed significant up-regulation in the gills, hepatopancreas and mantles after 21-day exposure to 2.5 mg/L TNT [124]. At a similar concentration, TNT induced the carbonyl reductase 1 gene (*Dma*_*CR1*) in *Daphnia magna* after 24-h exposure [44]. Oxidative stress can also lead to carbonyl stress; whereby ROS attack cellular carbonyl compounds to form reactive carbonyl species. Reactive carbonyl species formed can cumulate, damage macromolecules, and trigger further protein carbonylation, leading to cellular dysfunction [125]. Hence, the induction of carbonyl reductases (CR) is regarded as a significant cytoprotective response to OS [126,127]. Human CBR1 and Chinese hamster CR3 bear both xenobiotic and antioxidant response elements (XRE and ARE, respectively) in their promoter region, with the ARE serving as a target for Nrf2 binding [128,129]. Furthermore, CR1 substrates include the lipid peroxidation product 4-oxo-2-nonenal [130]. CBR1 induction via the Keap1/Nrf2/ARE signalling pathway has also been observed under OS conditions [131,132].

## 6 TNT mutagenicity and carcinogenicity

Regarding TNT's potential mutagenicity and carcinogenicity, scientific findings and classifications by expert bodies seem to differ. The International Agency for Research on Cancer (IARC), for example, categorises TNT as unclassifiable in terms of its carcinogenicity in humans (Group 3; (https://inchem.org/documents/iarc/vol65/trinitrotoluene.html), in spite of evidence from human studies. On the other hand, the United States Environmental Protection Agency (EPA) classifies TNT as a possible human carcinogen (Group C; (https://nepis.epa.gov/Exe/ZyPURL.cgi?Dockey=P100MWSU.txt); the Deutsche Forschungsgemeinschaft (DFG) considers TNT to be a human carcinogen (germ cell mutagenicity Cat 3 B) (https://www.baua.de). This disparity in classification shows that the evidence on the mutagenic and (especially) carcinogenic properties of TNT may not be sufficient to conclude on its classification. However, unlike the overall toxicity of TNT, which results from its metabolic activation (see discussions in previous Subsections), mutagenic and carcinogenic effects observed could be caused by both the parent compound and its metabolites (including more stable metabolites, such as 2-ADNT and 4-ADNT).

It has been known for decades that TNT, but not its metabolites, has a mutagenic effect in the bacterium *Salmonella typhimurium* [133]. In both TA98 and TA100 *S. typhimurium* strains, TNT mutagenicity was observed regardless of its metabolic activation [9]. The urine of TNT-exposed workers (exposure by inhalation and, probably, dermal) was reported, as early as 1985, to show a mutagenic effect in the TA98 *S. typhimurium* strain (without a metabolic activation system - S-9 mix) [134]. These samples could certainly contain urinary metabolites of TNT and, perhaps, some amounts of the parent compound (significant growth of revertant colonies occurred in the absence of nitroreductases (using TA 98 deficient of nitroreductases). Subsequently, several studies also showed that TA100 (strain for investigating base pair substitutions by xenobiotics) was more sensitive than TA98 (strain for investigating frameshift mutations) to organic matrices loaded with TNT [135,136]. Particularly, samples with unmetabolised TNT demonstrated significant back mutations. However, Sabbioni et al. [137] showed a correlation between the concentrations of 2-ADNT and 4-ADNT (principal TNT metabolites) in the urine of TNT-exposed workers and the occurrence of back mutations using the AMES test [137]. Both metabolites caused a concentration-dependent mutagenic effect in the bacteria.

Although the bacterial mutagenicity of TNT and its metabolites has been extensively investigated and demonstrated by several studies, inconsistencies remain, especially regarding some TNT metabolites. For example, studies by Lachance et al. showed that some of the metabolites only exhibit mutagenic effects after long exposure times or exposure to high concentrations, which generally indicates weak mutagenicity [136]. They also observed a mutagenic effect by the diamino metabolite 2,4-DANT. Tan et al. [138] observed a decline in mutagenicity with increasing formation of amino metabolites (due to reduction of nitro groups) [138]. They postulated that the presence of a nitro group at position 4 could account for the mutagenic properties of TNT metabolites. In general, metabolic detoxification seems to play a role, as the addition of S-9 mix usually led to a reduction in mutagenic effects. These findings (which are solely based on DNA modification by TNT and its metabolites in bacteria) were comparable to the higher sensitivity of Chinese hamster cells (V79) to explosives [136]. Kennel et al. [139] also reported TNT mutagenicity in ovarian cells from the Chinese hamster [139].

Furthermore, TNT carcinogenicity has been poorly defined over the years due to differences in species and tissues used in investigations as well as the lack of sufficient data. In 1990, Ryan and Ross reported hyperplasia in the liver and kidney, and carcinoma of the urinary bladder in Fisher 344 rats fed with TNT-containing diets (0.4–50 mg/kg) [140]. TNT also demonstrated higher genotoxicity than 2-ADNT and 4-ADNT in zebrafish embryos with significant tail DNA content [141]. The uncertainty regarding TNT carcinogenicity could also result from contrasting reports and varying exposure durations. For instance, urinary carcinoma was recorded after a two-year exposure of Fisher 344 rats to TNT through food [142], while short-term exposure (24–72 h) failed to elicit bone marrow toxicity [143]. Thus, persistent exposure to TNT and its metabolites could promote its carcinogenicity. The accumulation of TNT, and that of its metabolites, in organisms such as mussels [4,5,144] and fish [121,144] is therefore of ecological relevance.

One can infer that although TNT does not cause OS apart from its metabolic activation, the parent compound possesses significant mutagenic and carcinogenic properties. This could amplify its genotoxicity upon chronic exposure; genotoxic TNT metabolites (both reactive and stable) also build up with time. Furthermore, the cell type and types of metabolites formed could influence the extent of DNA damage observed. Genotoxicity of its reactive metabolites can, however, not be understated. Section 8 discusses the evidence on mutagenicity and carcinogenicity of TNT metabolites further.

## 7 TNT toxicities *in vivo*

Historically, occupational exposure to TNT is known to cause serious toxicities in humans. Following both acute and chronic exposure, TNT absorption (predominantly via skin and inhalation) results in contact dermatitis, hepatotoxicity, cataract and hematological disorders such as methemoglobinemia and aplastic anaemia [71,[145], [146], [147]].

### 7.1 Cataract

Although corneal absorption of TNT can be regarded as insignificant, it is not implausible and may contribute to the occurrence of cataract. The association of cataract with TNT dates back as early as 1932, when dark turbid rings were found to cover the pupil halfway after two to three years of TNT exposure in the Soviet Union [71,148]. Disease progression in these subjects was confirmed by the occurrence of spike-like opacities in the equator of the lens two to three years later [71,148]. Evidence indicates that TNT-induced cataracts do not affect visual acuity [149]. Hassman and Jaman [150] also reported cataract among 42% of 61 TNT workers after 8.4 years of occupational exposure [150]. Chronic TNT exposure to Finish workers saw 50% cases of cataracts [149]. Deterioration of the ocular antioxidant system also contributes to susceptibility of TNT-induced cataract, which is seems to increase with age [151,152].

TNT-induced cataract has been categorized as bilateral and symmetrical throughout its four stages of occurrence (defined by Tiukina [153]), with the onset of dust-like ring opacities in the equator of the lens progressing to dust-rings with triangular edges covering the centre of the lens [153]. It has become apparent that incidences of TNT-induced cataract are significantly influenced by intensity of exposure with the source of exposure playing little role. Cataract was observed after exposure to low concentrations of TNT [71,149]. This supports the hypothesis that ROS build-up and/or the formation of reactive TNT metabolites over time causes irreversible ocular damage (cataract in this case). The occurrence of ring-shaped cataracts in TNT workers correlated positively with the presence and accumulation of TNT and 2/4-ADNT, respectively in urine samples; 2,4-DNT and 2,6-DNT were also significantly recorded [74]. Interestingly, sporadic observations of increased ring-shaped cataract formation in old-aged TNT workers [149] seem to support the notion that age (which undoubtedly affects the integrity of ocular the antioxidant system) increases susceptibility to TNT-induced cataracts. This could be supported by the physiological predisposition of the lens to peroxidation. However, these reports are not consistent [74].

### 7.2 Hematotoxicity

TNT hematotoxicity presents as hemolysis and erythrocyte damage as well as increased methemoglobin (metHb) and sulfhemoglobin formation [154,155]. This is typical of nitroaromatics. Methemoglobinemia, which is generally characterised by an increase in cyanosis (above 1.5 g/dl) and methemoglobin saturation (10–30%), has been observed following both acute and chronic occupational exposure; among chemical industrial workers and miners [156]. TNT exposure following blast injury can also result in hematotoxicity in humans. Decreased hemoglobin (Hb) saturation, arterial partial oxygen pressure and arterial oxygen saturation, and metHb saturation of 18%, cyanosis and chocolate-coloured blood from blast following a TNT-containing bomb manipulation led to a methemogloninemia diagnosis [156]. Compared to other explosives such as tetryl and pentyl, TNT is a more potent metHb inducer [157].

TNT-induced anemia in beagle dogs caused a five-fold increase in metHb and approximately two-fold increase in urinary bilirubin (above 8 mg/kg TNT) with significant decrease in Hb and hematocrit (approximately 1.3- and 1.2-fold, respectively at 32 mg/kg TNT) after daily administration for six months [158]. In male Fischer 344 rats, oral intake of TNT (300 mg/kg/day) caused significant increase in metHb formation, while decreasing hematocrit levels after a four-day administration [146]. Subsequent *in vitro* investigations by these authors revealed that interactions between erythrocytes and the reactive metabolite HADNT resulted in these effects; 500 μM HADNT caused a two-fold increase in hemolysis compared to TNT. HADNT reactions with metHb and oxyhemoglobin (oxyHb) increased H_2_O_2_ formation resulting in significantly low Hb thiol content [146]. The reduction of Hb thiol content certainly promotes susceptibility of erythrocytes to ROS. Nitroaromatic-mediated erythrocyte GSH depletion creates prooxidant conditions (via conjugate formation) that bolster Hb oxidation to metHb [159]. Ferrylhemoglobin (ferrylHb) produced from reactions between oxyHb and HADNT (not TNT, ADNT or AADNT - 4-acetylamino-2,6-dinitrotoluene, an ADNT metabolite), in addition to H_2_O_2_ promote Hb aggregation via disulphide formation of globin moiety [146]. HADNT-induced H_2_O_2_ and metHb formation does not deviate from the erythrotoxic effect of hydroxylamine analogues in general, which is characterized by increase radical formation, lipid peroxidation, depletion of detoxification enzymes such as glutathione S-transferase and methemoglobin reductase, and oxidative stress via coupled redox reaction with oxyHb [160]. Furthermore, H_2_O_2_ on its own can increase metHb levels via oxidizing oxyHb [161], and oxidise ferrous and ferric hemoglobin to ferrylHb [162,163]. FerrylHb is cytotoxic and oxidative towards mitochondria (causing a reduction in mitochondrial membrane potential and mitochondrial dysfunction) [164]. HADNT triggers the release of heme from oxyHb leading to decreased Hb oxygen saturation and hemin release [146]. Hemin is a hemolytic agent which inhibits delta-aminolevinic acid (a heme precursor) [165,166] but induces the heme-degrading enzyme HO-1 [167]. Thus, HADNT mediates TNT-induced hemolysis and negative heme balance.

### 7.3 Contact dermatitis

Direct absorption of TNT via the skin leads to allergic contact dermatitis. This seems to occur irrespective of the duration of exposure, although dermatitis is often reported in chronic exposure [147]. Moreover, the intensity of exposure to TNT and TNT-containing materials influences the severity of skin damage. Some of the initial cases of dermatitis from TNT exposure revealed rapid onset of urticaria (associated with purpuric spots and petechiae) and vascular eruptions [168], yellowing of palms, fingers and nails with oedema, and erythematous rash [147,169,170]. Interestingly, remission of symptoms was reported when exposure to TNT was removed [147,169,170]. Dermatitis resulting from blast injuries is also known and often associated with more systemic effects such as cyanosis. The involvement of oxidative stress in the onset and progression of contact dermatitis highlights even further the potential role of reactive TNT metabolites and ROS generated from TNT reduction in TNT dermatitis [171]. Nevertheless, TNT as the parent compound may contribute more significantly to these dermatological irritations.

## 8 Significance of TNT metabolites: the gap in TNT toxicity and mutagenicity research

As previously discussed, TNT metabolism leads to the generation of prooxidative metabolites. Although TNT toxicity is relatively well-researched, the contribution of its reactive metabolites (and other metabolites) to these toxicities is vaguely understood. However, considering discussions in the previous Sections, it can be inferred that the formation, and in some cases build up, of reactive intermediates of TNT metabolism could account for a large proportion of TNT toxicity and mutagenicity. This hypothesis could be relevant when considering the long-term effects of TNT exposure.

The notion that TNT metabolites cause significant toxicities is irrefutable. In addition, urine from workers exposed to TNT was found to contain mutagenic components [134]. Bladder cancer was also observed in TNT-fed rats [9]; possibly due to the presence of carcinogenic TNT metabolites. Similarly, genotoxic effects observed in male Fischer 344 rats were caused by the N-hydroxy TNT metabolite HADNT [56]. Compared to 2,4-DNT, TNT was less effective in causing DNA single-strand breakage in rat hepatocytes [143]. Modulation of DNA damage and metabolic processes in rat hepatocytes by 2,4-DNT and 2,6-DNT was also more significant than the TNT effects [78]. Hence, it can be inferred that these findings resulted from DNA adducts formed by interactions between TNT metabolites and DNA molecules; thus, hindering DNA synthesis and replication [172]. Protein adducts were also formed in rat liver microsomes because of NADPH-dependent bioactivation of TNT to the reactive intermediate 4-hydroxylamino-2,6-DNT [173]. Mussels (*Mytilus galloprovincialis*) and fish (*Cyprinodon variegatus*) also retained protein adducts of aminodinitrotoluenes and diaminoni trotoluenes in their tissues [144]. Furthermore, TNT toxicity is also reflected in the formation of its biotransformation products such as arylhydroxylamine, arylamine, azoxy- and azo-derivatives [174].

On the contrary, evidence suggests that TNT could be more cytotoxic than its stable metabolites such as 2-ADNT, 4-ADNT, 2,4-DANT, and, 2,6-DANT [136]. In HepG2 cells, TNT was more cytotoxic, indicated by a decline in cell viability (LC_50_ = 105 ± 6 μg/ml) and HSP 70 and GRP78 induction compared to its by-products 2,4-DNT>2,6-DNT [77]. Acute toxicity studies conducted by Koske et al. [141] showed that TNT was three-fold more toxic in *Danio rerio* than its principal metabolites 2-ADNT and 4-ADNT (LC_50_ values: 4.5 mg/l, 13.4 mg/l and 14.4 mg/l, respectively) [141]. These observations corresponded positively with approximately 16% tail DNA formation by 0.1 mg/l TNT after 48 h exposure; 2-ADNT and 4-ADNT (at 1 mg/l) resulted in 12–13% tail DNA content. Again, it is noteworthy that this four-fold increase in TNT genotoxicity coefficient could have resulted from genotoxic effects of reactive intermediates such as 4-HADNT, nitrites, nitrates and other ROS, which are formed in the early stages of TNT reduction by NADPH Cytochrome-P450 reductase [68,141,175]. NOEC obtained from a five-day exposure in Juvenile sheepshead minnows (*Cyprinodon variegatus*) were 1.5 mg/l, 4.0 mg/l, and 56 mg/l for TNT, 2-ADNT, and 2,4-DANT, respectively [176]. Marine flatworms showed increased sensitivity to 2-ADNT and 4-ADNT than the parent compound; this was observed as suppressed feeding and a temperature-dependent rise in lethality [177].

However, the toxicity potential of other TNT intermediates could surpass that of the parent compound. An even stronger cytotoxic propensity is predicted for the –NHOH and –NH2 metabolites of TNT in accordance with their nucleophilic properties [[178], [179], [180]]. For example, the complete absence of nitro substituents (nitro groups are well-known toxicophores [13]) in the structures of TNT intermediates, such as 2,4,6-triaminotoluene (TAT), does not necessarily correspond to low toxicity. TAT, a late phase intermediate metabolite formed from nitroreduction of 2,4-DANT and 2,6-DANT was approximately 38-fold more cytotoxic than TNT in V79 Chinese hamster lung cells, with LOEC of 1 μM and 33 μM, respectively [136]. Cytochrome-P450-catalysed oxidation of such amino metabolites to their hydroxylamines could account for this enhanced cytotoxicity [181]. More so, 2NHOH-DNT and 2NH_2_-DNT recruit the ferocious ^•^OH radical to mediate their cytotoxic effects [60]. Depending on the cell type, the toxic effects of NHOH-DNTs could occur via redox cycling and DNA alkylating properties [55]. Although nitroreductase-catalysed two-electron reduction of 2-/4-hydroxylamino DNTs does not produce free radicals [60], autooxidation of 2,4-dihydroxyamino-6-nitrotoluene (a subsequent intermediate, which is also a substrate for final reduction) has been reported [182]. Hydroxylamino-dinitrotoluenes and their nitroso reoxidation products also interact with proteins via covalent binding [173]. Evidently, TNT toxicity (including its mutagenic and carcinogenic effects) cannot be discussed in isolation from its stable and unstable metabolites.

## 9 Relevance of OS markers in TNT biomonitoring and risk assessment

Further to reports on the assessment of cellular ROS levels and induction of classic antioxidant enzymes as a measure of TNT-induced OS, the carbonyl reductase gene has recently become of interest as a biomarker for TNT biomonitoring [6,44,124,183]. The assessment of protein carbonylation during OS reveals a rather severe or advanced phase of OS, where oxidation of cellular proteins alters protein structure (loss of sulfhydryl groups and realignment of amino acid resonance) and function [184]. The use of OS-assessment techniques such as TOSC [[185], [186], [187]] and comet assay [188] have also proved useful in ascertaining the toxic effect of ROS (which serve as effect biomarkers) by environmental pollutants. Therefore, OS assessment could prove useful in eco-monitoring of contaminated sites.

Common measures of OS induction by TNT have included activity and expression of antioxidant enzymes such as SOD, catalase, and glutathione peroxidase and reductase (which could be classified as ‘first response antioxidant defence’); the occurrence of ROS-damaging effects such as lipid peroxidation and oxidative DNA damage; and occasionally identification of reactive species generated after exposure. While induction of ‘first response antioxidant defence’ can indicate subtle redox changes caused by toxicants, these could be more appropriately categorized as response biomarkers and may not always reflect the extent of oxidative damage caused when assessed independently. This is because their induction can result in adaptive response to pro-oxidants via consumption of initial ROS formed; this could subsequently be reversed by an overwhelmed cellular antioxidant defence system. Thus, the occurrence of OS in an organism is strongly influenced by the nature of stressor assessed and the duration of exposure. Environmental toxicants such as TNT often occur in the environment at relatively low concentrations, and this promotes their persistence, leading to increasing concentrations as influenced by environmental factors. This is certainly the case for sea-dumped munitions. Thus, OS biomarkers which could depict persistent redox instability such as lipid peroxidation, protein carbonylation, and Nrf2 induction (the induction of indirect antioxidant enzymes; see Ref. [61,62] could be better suited as a realistic measure of ecological impact of environmental toxicants.

Environmental pollutants such as heavy metals [189] and other munition compounds are also known to induce oxidative stress. The occurrence of carcinogenic metals, including arsenic, lead, mercury, aluminum and titanium oxide, in contaminated sites can be traced to their use in ordnance casings [10]. Toxicity (and, in a wider scope, ecological relevance) of diverse mixtures of pharmaceuticals present in the environment has also been adequately defined based on their ability to induce OS in aquatic organisms [190,191]). The occurrence of these pollutants, together with munition compounds such as TNT, is a realistic prediction in recent times, and this has created a huge environmental awareness among researchers and the public alike. Therefore, an effective assessment of OS in mediating TNT toxicities cannot be done in isolation of their levels in pollutant sites, and their exposure accumulation or metabolism in exposed organisms (see Ref. [3,4,121,124]).

## 10 Conclusion

TNT toxicity is of concern to humans and the ecosystem at large due to its persistence in the environment. The role of oxidative stress in mediating TNT toxicities stems from the significant efforts of metabolizing enzymes, which in attempt to detoxify the recalcitrant compound, generate noxious reactive intermediates that compound its toxicity. More importantly, the reduction of its nitro groups to amino derivatives could also result in increased cytotoxicity as a result of ROS formed. Thus, TNT toxicity cannot be sufficiently defined without a comprehensive assessment of TNT metabolites formed, their subsequent metabolism and cellular effects, as well as the potential mutagenicity and carcinogenicity of the parent compound. However, the causative role of OS in mediating toxicities by environmental pollutants other than TNT can also not be overlooked. The presence of such co-stressors, including metal components of munition shells and other munition-related components, in TNT-contaminated areas is inevitable. Therefore, the use of OS (in addition to other detection methods for the assessment of TNT levels in contaminated areas) could be more effective as a biomonitoring tool in evaluating TNT remediation efforts as well as toxicity risks.

All authors have read and agreed to the published version of the manuscript.

## Declaration of data derived

I, as the corresponding author, declare that the work described here has not been published previously nor is under consideration for publication elsewhere. The publication is approved by all of the coauthors.

## CRediT authorship contribution statement

**Amma Gyapomah Adomako-Bonsu:** Writing – original draft, Visualization, Conceptualization. **Jana Jacobsen:** Writing – original draft, Visualization, Formal analysis, Data curation. **Edmund Maser:** Writing – review & editing, Supervision, Project administration, Investigation, Funding acquisition, Data curation, Conceptualization.

## Declaration of competing interest

I, as the corresponding author, declare on behalf of all the authors of the submission, that there is not any financial interest or personal relationship with other people or organizations that could inappropriately influence this work.

## Data Availability

No data was used for the research described in the article.

## References

[1] Rice A. (1918). Risk and avoidance of TNT poisoning. Mon. Rev. US Bur. Labor Stat..

[2] Janßen R., Beck A.J., Werner J., Dellwig O., Alneberg J., Kreikemeyer B., Maser E., Böttcher C., Achterberg E.P., Andersson A.F., Labrenz M. (2021). Machine learning predicts the presence of 2, 4, 6-trinitrotoluene in sediments of a baltic sea munitions dumpsite using microbial community compositions. Front. Microbiol..

[3] Strehse J.S., Maser E. (2020). Marine bivalves as bioindicators for environmental pollutants with focus on dumped munitions in the sea: a review. Mar. Environ. Res..

[4] Appel D., Strehse J.S., Martin H.-J., Maser E. (2018). Bioaccumulation of 2,4,6-trinitrotoluene (TNT) and its metabolites leaking from corroded munition in transplanted blue mussels (M. edulis). Mar. Pollut. Bull..

[5] Maser E., Strehse J.S. (2021). Can seafood from marine sites of dumped World War relicts be eaten?. Arch. Toxicol..

[6] Strehse J.S., Brenner M., Kisiela M., Maser E. (2020). The explosive trinitrotoluene (TNT) induces gene expression of carbonyl reductase in the blue mussel (Mytilus spp.): a new promising biomarker for sea dumped war relicts?. Arch. Toxicol..

[7] Yoo L.J., Lotufo G.R., Gibson A.B., Steevens J.A., Sims J.G. (2006). Toxicity and bioaccumulation of 2, 4, 6-trinitrotoluene in fathead minnow (Pimephales promelas). Environ. Toxicol. Chem..

[8] Maser E., Bünning T.H., Strehse J.S. (2023). Environmental and human toxicology studies on explosive chemicals leaking from submerged munitions. Propellants, Explos. Pyrotech..

[9] Bolt H.M., Degen G.H., Dorn S.B., Plöttner S., Harth V. (2006). Genotoxicity and potential carcinogenicity of 2,4,6-TNT trinitrotoluene: structural and toxicological considerations. Rev. Environ. Health.

[10] Sanderson H., Fauser P., Stauber R.S., Christensen J., Løfstrøm P., Becker T. (2017). Civilian exposure to munitions-specific carcinogens and resulting cancer risks for civilians on the Puerto Rican island of Vieques following military exercises from 1947 to 1998. Global Security: Health, Science and Policy.

[11] Čėnas N., Nemeikaitė-Čėnienė A., Kosychova L. (2021). Single-and two-electron reduction of nitroaromatic compounds by flavoenzymes: mechanisms and implications for cytotoxicity. Int. J. Mol. Sci..

[12] Kovacic P., Somanathan R. (2014). Nitroaromatic compounds: environmental toxicity, carcinogenicity, mutagenicity, therapy and mechanism. Appl. Toxicol..

[13] Nepali K., Lee H.-Y., Liou J.-P. (2018). Nitro-group-containing drugs. J. Med. Chem..

[14] Purohit V., Basu A.K. (2000). Mutagenicity of nitroaromatic compounds. Chem. Res. Toxicol..

[15] Singh D., Mishra K., Ramanthan G. (2015). Bioremediation of nitroaromatic compounds. Wastewater treatment engineering.

[16] Spain J.C. (1995). Biodegradation of nitroaromatic compounds. Annu. Rev. Microbiol..

[17] Haas R., Schreiber I., van Low E., Stork G. (1990). Conception for the investigation of contaminated munition plants: 2. Investigation of former RDX-plants and filling stations, Fresen. J. Anal. Chem..

[18] Sjӧstrӧm J., Karlsson R., Qvarfort U. (2004). FOI-R--1307--SE. Umeå.

[19] Juhasz A.L., Naidu R. (2007). Explosives: fate, dynamics, and ecological impact in terrestrial and marine environments. Rev. Environ. Contam. Toxicol..

[20] Yamamoto H., Morley M.C., Speitel G.E., Clausen J.A.Y. (2004). Fate and transport of high Explosives in a sandy soil: adsorption and desorption, soil and sediment contamination. Int. J..

[21] Elovitz M.S., Weber E.J. (1999). Sediment-mediated reduction of 2,4,6-trinitrotoluene and fate of the resulting aromatic (Poly)amines. J. Am. Chem. Soc..

[22] Prak D.J.L., Breuer J.E.T., Rios E.A., Jedlicka E.E., O'Sullivan D.W. (2017). Photolysis of 2, 4, 6-trinitrotoluene in seawater and estuary water: impact of pH, temperature, salinity, and dissolved organic matter. Mar. Pollut. Bull..

[23] Spain J.C., Hughes J.B., Knackmuss H.-J. (2000). Biodegradation of Nitroaromatic Compounds and Explosives.

[24] Lotufo G.R., Lydy M.J., Rorrer G.L., Cruz-Uribe O., Cheney D.P., Sunahara G.I., Lotufo G., Kuperman R.G., Hawari J. (2009). Ecotoxicology of Explosives.

[25] Johnson M.S., Salice C.J., Sample B.E., Robidoux P.Y., Sunahara G.I., Lotufo G., Kuperman R.G., Hawari J. (2009). Ecotoxicology of Explosives.

[26] Renoux A.Y., Sarrazin M., Hawari J., Sunahara G.I. (2009). Transformation of 2,4,6-trinitrotoluene in soil in the presence of the earthworm Eisenia andrei. Environ. Toxicol. Chem..

[27] Čėnas N., Aušra N., Henrikas N., Anusevičius Ž., Šarlauskas J. (2001). Quantitative structure–activity relationships in enzymatic single-electron reduction of nitroaromatic explosives: implications for their cytotoxicity. Biochim. Biophys. Acta BBA-Gen. Subj..

[28] Bryant C., DeLuca M. (1991). Purification and characterization of an oxygen-insensitive NAD (P) H nitroreductase from Enterobacter cloacae. J. Biol. Chem..

[29] Butler J., Hoey B.M. (1993). The one-electron reduction potential of several substrates can be related to their reduction rates by cytochrome P-450 reductase. Biochim. Biophys. Acta Protein Struct. Mol. Enzymol..

[30] Orna M.V., Mason R.P. (1989). Correlation of kinetic parameters of nitroreductase enzymes with redox properties of nitroaromatic compounds. J. Biol. Chem..

[31] Peterson F.J., Mason R.P., Hovsepian J., Holtzman J.L. (1979). Oxygen-sensitive and-insensitive nitroreduction by Escherichia coli and rat hepatic microsomes. J. Biol. Chem..

[32] Adams G.E., Clarke E.D., Jacobs R.S., Stratford I.J., Wallace R.G., Wardman P., Watts M.E. (1976). Mammalian cell toxicity of nitro compounds: dependence upon reduction potential. Biochem. Biophys. Res. Commun..

[33] Guissani A., Henry Y., Lougmani N., Hickel B. (1990). Kinetic studies of four types of nitroheterocyclic radicals by pulse radiolysis: correlation of pharmacological properteis to decay rates. Free Radic. Biol. Med..

[34] Miŝkinien V., Sergedien E., Nemeikait A., Segura-Aguilar J., Čėnas N. (1999). Role of redox cycling and activation by DT-diaphorase in the cytotoxicity of 5-(aziridin-1-yl)-2, 4-dinitrobenzamide (CB-1954) and its analogs. Cancer Lett..

[35] O’brien P.J., Wong W.C., Silva J., Khan S. (1990). Toxicity of nitrobenzene compounds towards isolated hepatocytes: dependence on reduction potential. Xenobiotica.

[36] Siim B.G., Atwell G.J., Wilson W.R. (1994). Metabolic and radiolytic reduction of 4-alkylamino-5-nitroquinoline bioreductive drugs: relationship to hypoxia-selective cytotoxicity. Biochem. Pharmacol..

[37] Finkel T. (2012). Signal transduction by mitochondrial oxidants. J. Biol. Chem..

[38] Zitting A., Szumańska G., Nickels J., Savolainen H. (1982). Acute toxic effects of trinitrotoluene on rat brain, liver and kidney: role of radical production. Arch. Toxicol..

[39] Esteve-Núñez A., Caballero A., Ramos J.L. (2001). Biological degradation of 2, 4, 6-trinitrotoluene. Microbiol. Mol. Biol. Rev..

[40] Johnston E.J., Rylott E.L., Beynon E., Lorenz A., Chechik V., Bruce N.C. (2015). Monodehydroascorbate reductase mediates TNT toxicity in plants. Science.

[41] Daun G., Lenke H., Reuss M., Knackmuss H.-J. (1998). Biological treatment of TNT-contaminated soil. 1. Anaerobic cometabolic reduction and interaction of TNT and metabolites with soil components. Environ. Sci. Technol..

[42] Hawari J., Beaudet S., Halasz A., Thiboutot S., Ampleman G. (2000). Microbial degradation of explosives: biotransformation versus mineralization. Appl. Microbiol. Biotechnol..

[43] Kumagai Y., Kikushima M., Nakai Y., Shimojo N., Kunimoto M. (2004). Neuronal nitric oxide synthase (NNOS) catalyzes one-electron reduction of 2, 4, 6-trinitrotoluene, resulting in decreased nitric oxide production and increased nNOS gene expression: implication for oxidative stress. Free Radic. Biol. Med..

[44] Jacobsen J., Adomako-Bonsu A.G., Maser E. (2022). Induction of carbonyl reductase 1 (CR1) gene expression in Daphnia magna by TNT, but not its key metabolites 2-ADNT and 4-ADNT. Chem. Biol. Interact..

[45] Hajos A.K.D., Winston G.W. (1991). Dinitropyrene nitroreductase activity of purified NAD (P) H-quinone oxidoreductase: role in rat liver cytosol and induction by Aroclor-1254 pretreatment. Carcinogenesis.

[46] Knox R.J., Friedlos F., Boland M.P. (1993). The bioactivation of CB 1954 and its use as a prodrug in antibody-directed enzyme prodrug therapy (ADEPT). Cancer Metastasis Rev..

[47] Nivinskas H., Koder R.L., Anusevičius Ž., Šarlauskas J., Miller A.-F., Čėnas N. (2001). Quantitative structure–activity relationships in two-electron reduction of nitroaromatic compounds by Enterobacter cloacae NAD (P) H: nitroreductase. Arch. Biochem. Biophys..

[48] Misevičienė L., Anusevičius Ž., Šarlauskas J., Čėnas N. (2006). Reduction of nitroaromatic compounds by NAD (P) H: quinone oxidoreductase (NQO1): the role of electron-accepting potency and structural parameters in the substrate specificity. Acta Biochim. Pol..

[49] Naumenko E.A., Naumov A.V., Suvorova E.S., Gerlach R., Ziganshin A.M., Lozhkin A.P., Silkin N.I., Naumova R.P. (2008). Participation of oxygen in the bacterial transformation of 2, 4, 6-trinitrotoluene. Biochem. Med..

[50] Naumenko E.A., Sibgatullina G.V., Mukhitov A.R., Rodionov A.A., Il’inskaya O.N., Naumova R.P. (2013). 2, 4, 6-trinitrotoluene as a trigger of oxidative stress in Fagopyrum tataricum callus cells. Russ. J. Plant Physiol..

[51] Ziganshin A.M., Ziganshina E.E., Byrne J., Gerlach R., Struve E., Biktagirov T., Rodionov A., Kappler A. (2015). Fe (III) mineral reduction followed by partial dissolution and reactive oxygen species generation during 2, 4, 6-trinitrotoluene transformation by the aerobic yeast Yarrowia lipolytica. Amb. Express.

[52] Kong L.Y., Jiang Q.G., Qu Q.S. (1989). Formation of superoxide radical and hydrogen peroxide enhanced by trinitrotoluene in rat liver, brain, kidney, and testicle in vitro and monkey liver in vivo. Biomed. Environ. Sci. BES.

[53] Liao H.-Y., Kao C.-M., Yao C.-L., Chiu P.-W., Yao C.-C., Chen S.-C. (2017). 2, 4, 6-trinitrotoluene induces apoptosis via ROS-regulated mitochondrial dysfunction and endoplasmic reticulum stress in HepG2 and Hep3B cells. Sci. Rep..

[54] Sun Y., Sumi D., Kumagai Y. (2006). Serine 1179 phosphorylation of endothelial nitric oxide synthase caused by 2, 4, 6-trinitrotoluene through PI3K/Akt signaling in endothelial cells. Toxicol. Appl. Pharmacol..

[55] Miliukiene V., Čėnas N. (2008). Cytotoxicity of nitroaromatic explosives and their biodegradation products in mice splenocytes: implications for their immunotoxicity. Z. Naturforsch. C Biosci..

[56] Homma-Takeda S., Hiraku Y., Ohkuma Y., Oikawa S., Murata M., Ogawa K., Iwamuro T., Li S., Sun G.F., Kumagai Y., Shimojo N., Kawanishi S. (2002). 2,4,6-Trinitrotoluene-induced reproductive toxicity via oxidative DNA damage by its metabolite. Free Radic. Res..

[57] Naumenko E.A., Ahlemeyer B., Baumgart‐Vogt E. (2017). Species‐specific differences in peroxisome proliferation, catalase, and SOD2 upregulation as well as toxicity in human, mouse, and rat hepatoma cells induced by the explosive and environmental pollutant 2, 4, 6‐trinitrotoluene. Environ. Toxicol..

[58] Sun Y., Iemitsu M., Shimojo Nobutake, Miyauchi T., Amamiya M., Sumi D., Hayashi T., Sun G., Shimojo Nobuhiro, Kumagai Y. (2005). 2, 4, 6-Trinitrotoluene inhibits endothelial nitric oxide synthase activity and elevates blood pressure in rats. Arch. Toxicol..

[59] Gao J.-J., Wang B., Peng R.-H., Li Z.-J., Xu J., Tian Y.-S., Yao Q.-H. (2021). Phytoremediation of multiple persistent pollutants co-contaminated soil by HhSSB transformed plant. Environ. Res..

[60] Šarlauskas J., Nemeikaite-Č A., Anusevičius Ž., Misevičien L., Julvez M.M., Medina M., Gomez-Moreno C., Čėnas N. (2004). Flavoenzyme-catalyzed redox cycling of hydroxylamino-and amino metabolites of 2, 4, 6-trinitrotoluene: implications for their cytotoxicity. Arch. Biochem. Biophys..

[61] Baird L., Dinkova-Kostova A.T. (2011). The cytoprotective role of the Keap1–Nrf2 pathway. Arch. Toxicol..

[62] Dinkova‐Kostova A.T., Talalay P. (2008). Direct and indirect antioxidant properties of inducers of cytoprotective proteins. Mol. Nutr. Food Res..

[63] Itoh K., Tong K.I., Yamamoto M. (2004). Molecular mechanism activating Nrf2–Keap1 pathway in regulation of adaptive response to electrophiles. Free Radic. Biol. Med..

[64] Kültz D. (2005). Molecular and evolutionary basis of the cellular stress response. Annu. Rev. Physiol..

[65] Gajewska J., Chełchowska M., Rychłowska-Pruszyńska M., Klepacka T., Ambroszkiewicz J. (2022). Oxidative and antioxidative status expressed as OSI index and GSH/GSSG ratio in children with bone tumors after anticancer therapy completion. J. Clin. Med..

[66] Zitka O., Skalickova S., Gumulec J., Masarik M., Adam V., Hubalek J., Trnkova L., Kruseova J., Eckschlager T., Kizek R. (2012). Redox status expressed as GSH: GSSG ratio as a marker for oxidative stress in paediatric tumour patients. Oncol. Lett..

[67] Yakovleva G., Kurdy W., Gorbunova A., Khilyas I., Lochnit G., Ilinskaya O. (2022). Bacillus pumilus proteome changes in response to 2, 4, 6-trinitrotoluene-induced stress. Biodegradation.

[68] Kumagai Y., Wakayama T., Li S., Shinohara A., Iwamatsu A., Sun G., Shimojo N. (2000). ζ‐Crystallin catalyzes the reductive activation of 2, 4, 6‐trinitrotoluene to generate reactive oxygen species: a proposed mechanism for the induction of cataracts. FEBS Lett..

[69] Spector A. (1995). Oxidative stress‐induced cataract: mechanism of action. Faseb. J..

[70] Srivastava S.K., Ansari N.H., Bhatnagar A. (1990). Sugar induced cataractogenesis: a paradigm of oxidative tissue pathology?. Lens Eye Toxic. Res..

[71] Kruse A., Hertel M., Hindsholm M., Viskum S. (2005). Trinitrotoluene (TNT)‐induced cataract in Danish arms factory workers. Acta Ophthalmol. Scand..

[72] Lewis-Younger C.R., Mamalis N., Egger M.J., Wallace D.O., Lu C. (2000). Lens opacifications detected by slitlamp biomicroscopy are associated with exposure to organic nitrate explosives. Arch. Ophthalmol..

[73] Liu Y.-Y., Yao M., Fang J.-L., Wang Y.-W. (1995). Monitoring human risk and exposure to trinitrotoluene (TNT) using haemoglobin adducts as biomarkers. Toxicol. Lett..

[74] Sarwat S., Akhtar A., Dayan S.F.A., Shaheen N., Durrani H.S., Usman T., Afghani T. (2022). Ring-shaped cataract and urinary metabolites among 2, 4, 6-trinitrotoluene exposed population of Pakistan. Int. Ophthalmol..

[75] Zhou A.S. (1990). A clinical study of trinitrotoluene cataract. Pol. J. Occup. Med..

[76] Zhu Z., Li Z., Mi F., Lian S., Dong P., Wu Y., Sun X. (2002). Study on the relationship between the opacity of lens and the levels of 2, 6-dinitro-4-amino-toluene (DNAT) in the urine of workers exposed to trinitrotoluene (TNT). Zhonghua Lao dong wei sheng zhi ye bing za zhi zhonghua laodong weisheng zhiyebing zazhi chin. J. Ind. Hyg. Occup. Dis..

[77] Tchounwou P.B., Wilson B.A., Ishaque A.B., Schneider J. (2001). Transcriptional activation of stress genes and cytotoxicity in human liver carcinoma cells (HepG2) exposed to 2, 4, 6‐trinitrotoluene, 2, 4‐dinitrotoluene, and 2, 6‐dinitrotoluene. Environ. Toxicol. Int. J..

[78] Deng Y., Meyer S.A., Guan X., Escalon B.L., Ai J., Wilbanks M.S., Welti R., Garcia-Reyero N., Perkins E.J. (2011). Analysis of common and specific mechanisms of liver function affected by nitrotoluene compounds. PLoS One.

[79] Fourquet S., Guerois R., Biard D., Toledano M.B. (2010). Activation of NRF2 by nitrosative agents and H2O2 involves KEAP1 disulfide formation. J. Biol. Chem..

[80] Okazaki S., Tachibana T., Naganuma A., Mano N., Kuge S. (2007). Multistep disulfide bond formation in Yap1 is required for sensing and transduction of H_2_O_2_ stress signal. Mol. Cell.

[81] Ashino T., Yamanaka R., Yamamoto M., Shimokawa H., Sekikawa K., Iwakura Y., Shioda S., Numazawa S., Yoshida T. (2008). Negative feedback regulation of lipopolysaccharide-induced inducible nitric oxide synthase gene expression by heme oxygenase-1 induction in macrophages. Mol. Immunol..

[82] Eggler A.L., Small E., Hannink M., Mesecar A.D. (2009). Cul3-mediated Nrf2 ubiquitination and antioxidant response element (ARE) activation are dependent on the partial molar volume at position 151 of Keap1. Biochem. J..

[83] Suzuki T., Muramatsu A., Saito R., Iso T., Shibata T., Kuwata K., Kawaguchi S., Iwawaki T., Adachi S., Suda H. (2019). Molecular mechanism of cellular oxidative stress sensing by Keap1. Cell Rep..

[84] Holland R., Hawkins A.E., Eggler A.L., Mesecar A.D., Fabris D., Fishbein J.C. (2008). Prospective type 1 and type 2 disulfides of Keap1 protein. Chem. Res. Toxicol..

[85] Suzuki T., Yamamoto M. (2017). Stress-sensing mechanisms and the physiological roles of the Keap1–Nrf2 system during cellular stress. J. Biol. Chem..

[86] Hogg N. (2002). The biochemistry and physiology of S-nitrosothiols. Annu. Rev. Pharmacol. Toxicol..

[87] Liu Z., Rudd M.A., Freedman J.E., Loscalzo J. (1998). S-Transnitrosation reactions are involved in the metabolic fate and biological actions of nitric oxide. J. Pharmacol. Exp. Therapeut..

[88] Martínez-Ruiz A., Lamas S. (2007). Signalling by NO-induced protein S-nitrosylation and S-glutathionylation: convergences and divergences. Cardiovasc. Res..

[89] Rossi R., Lusini L., Giannerini F., Giustarini D., Lungarella G., Di Simplicio P. (1997). A method to study kinetics of transnitrosation with nitrosoglutathione: reactions with hemoglobin and other thiols. Anal. Biochem..

[90] Wink D.A., Darbyshire J.F., Nims R.W., Saavedra J.E., Ford P.C. (1993). Reactions of the bioregulatory agent nitric oxide in oxygenated aqueous media: determination of the kinetics for oxidation and nitrosation by intermediates generated in the nitric oxide/oxygen reaction. Chem. Res. Toxicol..

[91] Kensler T.W., Wakabayashi N., Biswal S. (2007). Cell survival responses to environmental stresses via the Keap1-Nrf2-ARE pathway. Annu. Rev. Pharmacol. Toxicol..

[92] Rawat A., Gust K.A., Deng Y., Garcia-Reyero N., Quinn M.J., Johnson M.S., Indest K.J., Elasri M.O., Perkins E.J. (2010). From raw materials to validated system: the construction of a genomic library and microarray to interpret systemic perturbations in Northern bobwhite. Physiol. Genom..

[93] Fernández-Fernández M.R., Valpuesta J.M. (2018). Hsp70 chaperone: a master player in protein homeostasis. F1000Research.

[94] Zhang H., Gong W., Wu S., Perrett S. (2022). Hsp70 in redox homeostasis. Cells.

[95] Guo S., Wharton W., Moseley P., Shi H. (2007). Heat shock protein 70 regulates cellular redox status by modulating glutathione-related enzyme activities. Cell Stress Chaperones.

[96] Khomenko I., Bakhtina L.Y., Zelenina O., Kruglov S., Manukhina E., Bayda L., Malyshev I.Y. (2007). Role of heat shock proteins HSP70 and HSP32 in the protective effect of adaptation of cultured HT22 hippocampal cells to oxidative stress. Bull. Exp. Biol. Med..

[97] Gong P., Donohue K.B., Mayo A.M., Wang Y., Hong H., Wilbanks M.S., Barker N.D., Guan X., Gust K.A. (2018). Comparative toxicogenomics of three insensitive munitions constituents 2,4-dinitroanisole, nitroguanidine and nitrotriazolone in the soil nematode Caenorhabditis elegans. BMC Syst. Biol..

[98] Harrison P.M., Arosio P. (1996). The ferritins: molecular properties, iron storage function and cellular regulation. Biochim. Biophys. Acta BBA-Bioenerg..

[99] Rucker P., Torti F.M., Torti S.V. (1996). Role of H and L subunits in mouse ferritin. J. Biol. Chem..

[100] Epsztejn S., Glickstein H., Picard V., Slotki I.N., Breuer W., Beaumont C., Cabantchik Z.I. (1999). H-ferritin subunit overexpression in erythroid cells reduces the oxidative stress response and induces multidrug resistance properties. Blood J. Am. Soc. Hematol..

[101] Orino K., Lehman L., Tsuji Y., Ayaki H., Torti S.V., Torti F.M. (2001). Ferritin and the response to oxidative stress. Biochem. J..

[102] Peng T.-I., Jou M.-J. (2010). Oxidative stress caused by mitochondrial calcium overload. Ann. N. Y. Acad. Sci..

[103] Grover A.K., Samson S.E., Fomin V.P. (1992). Peroxide inactivates calcium pumps in pig coronary artery. Am. J. Physiol. Heart Circ. Physiol..

[104] Doan T.N., Gentry D.L., Taylor A.A., Elliott S.J. (1994). Hydrogen peroxide activates agonist-sensitive Ca^2+^-flux pathways in canine venous endothelial cells. Biochem. J..

[105] Graier W.F., Hoebel B.G., Paltauf-Doburzynska J., Kostner G.M. (1998). Effects of superoxide anions on endothelial Ca2+ signaling pathways. Arterioscler. Thromb. Vasc. Biol..

[106] Grover A.K., Samson S.E. (1988). Effect of superoxide radical on Ca^2+^ pumps of coronary artery. Am. J. Physiol. Cell Physiol..

[107] Ermak G., Davies K.J.A. (2002). Calcium and oxidative stress: from cell signaling to cell death. Mol. Immunol..

[108] Bernardi P., Azzone G.F. (1983). Regulation of Ca^2+^ efflux in rat liver mitochondria: role of membrane potential. Eur. J. Biochem..

[109] Catisti R., Vercesi A.E. (1999). The participation of pyridine nucleotides redox state and reactive oxygen in the fatty acid-induced permeability transition in rat liver mitochondria. FEBS Lett..

[110] Hunter D.R., Haworth R.A. (1979). The Ca^2+^-induced membrane transition in mitochondria: III. Transitional Ca^2+^ release. Arch. Biochem. Biophys..

[111] Jurkowitz M.S., Geisbuhler T., Jung D.W., Brierley G.P. (1983). Ruthenium red-sensitive and-insensitive release of Ca^2+^ from uncoupled heart mitochondria. Arch. Biochem. Biophys..

[112] Peng C.-F., Straub K.D., Kane J.J., Murphy M.L., Wadkins C.L. (1977). Effects of adenine nucleotide translocase inhibitors on dinitrophenol-induced Ca^2+^ efflux from pig heart mitochondria. Biochim. Biophys. Acta BBA-Bioenerg..

[113] Vercesi A.E. (1987). The participation of NADP, the transmembrane potential and the energy-linked NAD (P) transhydrogenase in the process of Ca^2+^ efflux from rat liver mitochondria. Arch. Biochem. Biophys..

[114] Zago E.B., Castilho R.F., Vercesi A.E. (2000). The redox state of endogenous pyridine nucleotides can determine both the degree of mitochondrial oxidative stress and the solute selectivity of the permeability transition pore. FEBS Lett..

[115] Kowaltowski A.J., Castilho R.F., Vercesi A.E. (2001). Mitochondrial permeability transition and oxidative stress. FEBS Lett..

[116] Korshunov S.S., Skulachev V.P., Starkov A.A. (1997). High protonic potential actuates a mechanism of production of reactive oxygen species in mitochondria. FEBS Lett..

[117] Fau D., Berson A., Eugene D., Fromenty B., Fisch C., Pessayre D. (1992). Mechanism for the hepatotoxicity of the antiandrogen, nilutamide. Evidence suggesting that redox cycling of this nitroaromatic drug leads to oxidative stress in isolated hepatocytes. J. Pharmacol. Exp. Therapeut..

[118] Pereira C.V., Nadanaciva S., Oliveira P.J., Will Y. (2012). The contribution of oxidative stress to drug-induced organ toxicity and its detection in vitro and in vivo. Expet Opin. Drug Metabol. Toxicol..

[119] Singh B.K., Tripathi M., Pandey P.K., Kakkar P. (2010). Nimesulide aggravates redox imbalance and calcium dependent mitochondrial permeability transition leading to dysfunction in vitro. Toxicology.

[120] Boelsterli U.A. (2002). Mechanisms of NSAID-induced hepatotoxicity: focus on nimesulide. Drug Saf..

[121] Beck A.J., Gledhill M., Kampmeier M., Feng C., Schlosser C., Greinert J., Achterberg E.P. (2022). Explosives compounds from sea-dumped relic munitions accumulate in marine biota. Sci. Total Environ..

[122] Edwards M., Bełdowski J. (2016). Chemical munitions dumped at sea. Deep Sea Res. Part II Top. Stud. Oceanogr..

[123] Koske D., Straumer K., Goldenstein N.I., Hanel R., Lang T., Kammann U. (2020). First evidence of explosives and their degradation products in dab (Limanda limanda L.) from a munition dumpsite in the Baltic Sea. Mar. Pollut. Bull..

[124] Strehse J.S., Appel D., Geist C., Martin H.-J., Maser E. (2017). Biomonitoring of 2,4,6-trinitrotoluene and degradation products in the marine environment with transplanted blue mussels (M. edulis). Toxicology.

[125] Heidari H., Kamalinejad M., Noubarani M., Rahmati M., Jafarian I., Adiban H., Eskandari M.R. (2016). Protective mechanisms of Cucumis sativus in diabetes-related modelsof oxidative stress and carbonyl stress. BioImpacts BI.

[126] Ebert B., Kisiela M., Malátková P., El-Hawari Y., Maser E. (2010). Regulation of human carbonyl reductase 3 (CBR3; SDR21C2) expression by Nrf2 in cultured cancer cells. Biochemistry.

[127] Rashid M.A., Lee S., Tak E., Lee J., Choi T.G., Lee J.-W., Kim J.B., Youn J.H., Kang I., Ha J. (2010). Carbonyl reductase 1 protects pancreatic β-cells against oxidative stress-induced apoptosis in glucotoxicity and glucolipotoxicity. Free Radic. Biol. Med..

[128] Miura T., Taketomi A., Nakabayashi T., Nishinaka T., Terada T. (2015). Identification of a functional antioxidant responsive element in the promoter of the Chinese hamster carbonyl reductase 3 (Chcr3) gene. Cell Biol. Int..

[129] Miura T., Taketomi A., Nishinaka T., Terada T. (2013). Regulation of human carbonyl reductase 1 (CBR1, SDR21C1) gene by transcription factor Nrf2. Chem. Biol. Interact..

[130] Martin H.-J., Ziemba M., Kisiela M., Botella J.A., Schneuwly S., Maser E. (2011). The Drosophila carbonyl reductase sniffer is an efficient 4-oxonon-2-enal (4ONE) reductase. Chem. Biol. Interact..

[131] Kwon J.H., Lee J., Kim J., Kirchner V.A., Jo Y.H., Miura T., Kim N., Song G.-W., Hwang S., Lee S.-G. (2019). Upregulation of carbonyl reductase 1 by Nrf2 as a potential therapeutic intervention for ischemia/reperfusion injury during liver transplantation. Mol. Cell..

[132] Yun M., Choi A.J., Lee Y.C., Kong M., Sung J.-Y., Kim S.S., Eun Y.-G. (2018). Carbonyl reductase 1 is a new target to improve the effect of radiotherapy on head and neck squamous cell carcinoma. J. Exp. Clin. Cancer Res..

[133] Won W.D., Disalvo L.H., Ng J. (1976). Toxicity and mutagenicity of 2, 4,-6-trinitrotoluene and its microbial metabolites. Appl. Environ. Microbiol..

[134] Ahlborg G., Einistö P., Sorsa M. (1988). Mutagenic activity and metabolites in the urine of workers exposed to trinitrotoluene (TNT). Occup. Environ. Med..

[135] Berthe-Corti L., Jacobi H., Kleihauer S., Witte I. (1998). Cytotoxicity and mutagenicity of a 2, 4, 6-trinitrotoluene (TNT) and hexogen contaminated soil in S. typhimurium and mammalian cells. Chemosphere.

[136] Lachance B., Robidoux P.Y., Hawari J., Ampleman G., Thiboutot S., Sunahara G.I. (1999). Cytotoxic and genotoxic effects of energetic compounds on bacterial and mammalian cells in vitro. Mutat. Res..

[137] Sabbioni G., Sepai O., Norppa H., Yan H., Hirvonen A., Zheng Y., Järventaus H., Bäck B., Brooks L.R., Warren S.H. (2007). Comparison of biomarkers in workers exposed to 2, 4, 6-trinitrotoluene. Biomarkers.

[138] Tan E.L., Ho C.H., Griest W.H., Tyndall R.L. (1992). Mutagenicity of trinitrotoluene and its metabolites formed during composting. J. Toxicol. Environ. Health Part Curr. Issues.

[139] Kennel S.J., Foote L.J., Morris M., Vass A.A., Griest W.H. (2000). Mutation analyses of a series of TNT‐related compounds using the CHO‐hprt assay. J. Appl. Toxicol. Int. J..

[140] Ryon M.G., Ross R.H. (1990). Water quality criteria for 2, 4, 6-trinitrotoluene. Regul. Toxicol. Pharmacol..

[141] Koske D., Goldenstein N.I., Kammann U. (2019). Nitroaromatic compounds damage the DNA of zebrafish embryos (Danio rerio). Aquat. Toxicol..

[142] Furedi E.M., Levine B.S., Gordon D.E., Rac V.S., Lish P.M. (1984).

[143] Ashby J., Burlinson B., Lefevre P., Topham J. (1985). Non-genotoxicity of 2, 4, 6-trinitrotoluene (TNT) to the mouse bone marrow and the rat liver: implications for its carcinogenicity. Arch. Toxicol..

[144] Lotufo G.R., Belden J.B., Fisher J.C., Chen S.F., Mowery R.A., Chambliss C.K., Rosen G. (2016). Accumulation and depuration of trinitrotoluene and related extractable and nonextractable (bound) residues in marine fish and mussels. Environ. Pollut..

[145] Sabbioni G., Liu Y.-Y., Yan H., Sepai O. (2005). Hemoglobin adducts, urinary metabolites and health effects in 2, 4, 6-trinitrotoluene exposed workers. Carcinogenesis.

[146] Shinkai Y., Li S., Kikuchi T., Kumagai Y. (2015). Participation of metabolic activation of 2, 4, 6-trinitrotoluene to 4-hydroxylamino-2, 6-dinitrotoluene in hematotoxicity. J. Toxicol. Sci..

[147] Williams H. (2007). Contact dermatitis within the explosives industry-a case report: allergies in the workplace. Curr. Allergy Clin. Immunol..

[148] Glezerov S.I. (1953). Katarakty of trinitrotoluola [Cataracts due to trinitrotoluene]. Vestn. Oftalmol..

[149] Härkönen H., Kärki M., Lahti A., Savolainen H. (1983). Early equatorial cataracts in workers exposed to trinitrotoluene. Am. J. Ophthalmol..

[150] Hassman P., Juran J. (1968). Cataract in persons working with trinitrotoluene (TNT). Int. Arch. Gewerbepathol. Gewerbehyg..

[151] Lou M.F. (2003). Redox regulation in the lens. Prog. Retin. Eye Res..

[152] Lou M.F., Dickerson J.E. (1992). Protein-thiol mixed disulfides in human lens. Exp. Eye Res..

[153] Tiukina G.A. (1967). Some features specific to the clinical picture of trinitrotoluene induced cataracts. Vestn. Oftalmol..

[154] Djerassi L.S., Vitany L. (1975). Haemolytic episode in G6 PD deficient workers exposed to TNT. Occup. Environ. Med..

[155] Levine B.S., Furedi E.M., Gordon D.E., Lish P.M., Barkley J.J. (1984). Subchronic toxicity of trinitrotoluene in Fischer 344 rats rats. Toxicology.

[156] Yazbeck-Karam V.G., Aouad M.T., Kaddoum R.N., Baraka A.S. (2004). Methemoglobinemia after a blast injury. J. Am. Soc. Anesthesiol..

[157] Marozienė A., Kliukienė R., Šarlauskas J., Čėnas N. (2001). Methemoglobin formation in human erythrocytes by nitroaromatic explosives. Z. Naturforsch. C Biosci..

[158] Levine B.S., Rust J.H., Barkley J.J., Furedi E.M., Lish P.M. (1990). Six month oral toxicity study of trinitrotoluene in beagle dogs. Toxicology.

[159] Awasthi Y.C., Garg H.S., Dao D.D., Partridge C.A., Srivastava S.K. (1981). Enzymatic conjugation of erythrocyte glutathione with 1-chloro-2, 4-dinitrobenzene: the fate of glutathione conjugate in erythrocytes and the effect of glutathione depletion on hemoglobin. Blood.

[160] Spooren A.A.M.G., Evelo C.T.A. (2000). A study on the interaction between hydroxylamine analogues and oxyhemoglobin in intact erythrocytes. Blood Cells. Mol. Dis..

[161] Kanais T., Acker J.P. (2010). Biopreservation of red blood cells – the struggle with hemoglobin oxidation. FEBS J..

[162] Dei Zotti F., Verdoy R., Brusa D., Lobysheva I.I., Balligand J.-L. (2020). Redox regulation of nitrosyl-hemoglobin in human erythrocytes. Redox Biol..

[163] Rifkind J.M., Nagababu E. (2013). Hemoglobin redox reactions and red blood cell aging. Antioxidants Redox Signal..

[164] Chintagari N.R., Jana S., Alayash A.I. (2016). Oxidized ferric and ferryl forms of hemoglobin trigger mitochondrial dysfunction and injury in alveolar type I cells. Am. J. Respir. Cell Mol. Biol..

[165] Gallo R.C. (1967). The inhibitory effect of heme on heme formation in vivo: possible mechanism for the regulation of hemoglobin synthesis. J. Clin. Invest..

[166] Karibian D., London I.M. (1965). Control of heme synthesis by feedback inhibition. Biochem. Biophys. Res. Commun..

[167] Tenhunen R., Zitting A., Nickels J., Savolainen H. (1984). Trinitrotoluene-induced effects on rat heme metabolism. Exp. Mol. Pathol..

[168] Preston J.F., Watkins C.A. (1946). Urticaria due to trinitrotoluene. Arch. Dermatol. Syphilol..

[169] Goh C.L., Rajan V.S. (1983). Contact sensitivity to trinitrotoluene. Contact Dermatitis.

[170] Goh C.L. (1984). Allergic contact dermatitis from tetryl and trinitrotoluene. Contact Dermatitis.

[171] Kaur S., Zilmer K., Leping V., Zilmer M. (2014). Allergic contact dermatitis is associated with significant oxidative stress. Dermatol. Res. Pract..

[172] Bermudez E., Tillery D., Butterworth B.E. (1979). The effect of 2, 4‐diaminotoluene and isomers of dinitrotoluene on unscheduled DNA synthesis in primary rat hepatocytes. Environ. Mutagen..

[173] Leung K.H., Yao M., Stearns R., Chiu S.-H.L. (1995). Mechanism of bioactivation and covalent binding of 2, 4, 6-trinitrotoluene. Chem. Biol. Interact..

[174] Rodgers J.D., Bunce N.J. (2001). Treatment methods for the remediation of nitroaromatic explosives. Water Res..

[175] Shinkai Y., Nishihara Y., Amamiya M., Wakayama T., Li S., Kikuchi T., Nakai Y., Shimojo N., Kumagai Y. (2016). NADPH-cytochrome P450 reductase-mediated denitration reaction of 2, 4, 6-trinitrotoluene to yield nitrite in mammals. Free Radic. Biol. Med..

[176] Lotufo G.R., Lydy M.J. (2005). Comparative toxicokinetics of explosive compounds in sheepshead minnows. Arch. Environ. Contam. Toxicol..

[177] Bickmeyer U., Meinen I., Meyer S., Kröner S., Brenner M. (2020). Fluorescence measurements of the marine flatworm Macrostomum lignano during exposure to TNT and its derivatives 2-ADNT and 4-ADNT. Mar. Environ. Res..

[178] Helsby N.A., Wheeler S.J., Pruijn F.B., Palmer B.D., Yang S., Denny W.A., Wilson W.R. (2003). Effect of nitroreduction on the alkylating reactivity and cytotoxicity of the 2, 4-dinitrobenzamide-5-aziridine CB 1954 and the corresponding nitrogen mustard SN 23862: distinct mechanisms of bioreductive activation. Chem. Res. Toxicol..

[179] Tercel M., Atwell G.J., Yang S., Stevenson R.J., Botting K.J., Boyd M., Smith E., Anderson R.F., Denny W.A., Wilson W.R. (2009). Hypoxia-activated prodrugs: substituent effects on the properties of nitro seco-1, 2, 9, 9a-tetrahydrocyclopropa [c] benz [e] indol-4-one (nitroCBI) prodrugs of DNA minor groove alkylating agents. J. Med. Chem..

[180] Wilson W.R., Stribbling S.M., Pruijn F.B., Syddall S.P., Patterson A.V., Liyanage H.S., Smith E., Botting K.J., Tercel M. (2009). Nitro-chloromethylbenzindolines: hypoxia-activated prodrugs of potent adenine N 3 DNA minor groove alkylators. Mol. Cancer Therapeut..

[181] Kim D., Kadlubar F.F., Teitel C.H., Guengerich P.F. (2004). Formation and reduction of aryl and heterocyclic nitroso compounds and significance in the flux of hydroxylamines. Chem. Res. Toxicol..

[182] Fiorella P.D., Spain J.C. (1997). Transformation of 2, 4, 6-trinitrotoluene by Pseudomonas pseudoalcaligenes JS52. Appl. Environ. Microbiol..

[183] Ebert B., Ebert D., Koebsch K., Maser E., Kisiela M. (2018). Carbonyl reductases from Daphnia are regulated by redox cycling compounds. FEBS J..

[184] Parvez S., Raisuddin S. (2005). Protein carbonyls: novel biomarkers of exposure to oxidative stress-inducing pesticides in freshwater fish Channa punctata (Bloch). Environ. Toxicol. Pharmacol..

[185] Iji O.T., Serem J.C., Bester M.J., Venter E.A., Myburgh J.G., McGaw L.J. (2017). Generation of reactive oxygen species in relevant cell lines as a bio-indicator of oxidative effects caused by acid mine water. WaterSA.

[186] Regoli F. (2000). Total oxyradical scavenging capacity (TOSC) in polluted and translocated mussels: a predictive biomarker of oxidative stress. Aquat. Toxicol..

[187] Regoli F., Gorbi S., Frenzilli G., Nigro M., Corsi I., Focardi S., Winston G.W. (2002). Oxidative stress in ecotoxicology: from the analysis of individual antioxidants to a more integrated approach. Mar. Environ. Res..

[188] Ge W., Yan S., Wang Jinhua, Zhu L., Chen A., Wang Jun (2015). Oxidative stress and DNA damage induced by imidacloprid in zebrafish (Danio rerio). J. Agric. Food Chem..

[189] Valko M.M.H.C.M., Morris H., Cronin M.T.D. (2005). Metals, toxicity and oxidative stress. Curr. Med. Chem..

[190] Nava-Álvarez R., Razo-Estrada A.C., García-Medina S., Gómez-Olivan L.M., Galar-Martinez M. (2014). Oxidative stress induced by mixture of diclofenac and acetaminophen on common carp (Cyprinus carpio). Water. Air. Soil Pollut.

[191] Stancova V., Plhalova L., Blahova J., Zivna D., Bartoskova M., Siroka Z., Marsalek P., Svobodova Z. (2017). Effects of the pharmaceutical contaminants ibuprofen, diclofenac, and carbamazepine alone, and in combination, on oxidative stress parameters in early life stages of tench (Tinca tinca). Vet. Med..

